# Pay-to-stay drives the evolution of helping independent of kin selection in anemonefish societies

**DOI:** 10.1093/beheco/arag061

**Published:** 2026-06-10

**Authors:** Kirstin Gaffney, Megan E Bartlett, Theresa Rueger

**Affiliations:** Dove Marine Laboratory, School of Natural and Environmental Science, Newcastle University, Cullercoats, North Shields, NE30 4PZ, United Kingdom; Dove Marine Laboratory, School of Natural and Environmental Science, Newcastle University, Cullercoats, North Shields, NE30 4PZ, United Kingdom; Dove Marine Laboratory, School of Natural and Environmental Science, Newcastle University, Cullercoats, North Shields, NE30 4PZ, United Kingdom

**Keywords:** social evolution, cooperation, helping, punishment, appeasement, pay-to-stay, marine fish, coral reefs

## Abstract

The evolutionary drivers of helping behavior continue to be widely debated, particularly due to challenges in disentangling the effects of different mechanisms. The pay-to-stay hypothesis proposes that helping aims to appease dominant breeders to avoid eviction. We tested pay-to-stay as an independent driver of helping by exploring its dynamics in anemonefishes (*Amphiprion spp.*) which lack prerequisites for other mechanisms, including kin selection. We experimentally prevented helping (anemone maintenance, territory defence) in *A. percula*, *A. perideraion*, and *A. clarkii*., and assessed 2 pay-to-stay predictions: (i) helpers would be punished and/or (ii) would compensate with increased cooperative effort (pre-emptive appeasement). We show that anemonefish nonbreeders do pay-to-stay: punishment and/or appeasement occurred in all species, with differences reflecting variation in ecological and social constraints. We also highlight a novel trade-off between size and cooperative effort in pay-to-stay dynamics. Overall, this study enhances our understanding of helping evolution, demonstrating that pay-to-stay can drive helping independently of kin selection.

## Teaser

We found evidence for “pay-to-stay” in anemonefishes (gaining future benefits from current cooperation at their own expense). This confirms that helping can evolve independently of high relatedness and be maintained solely through direct benefits. This contributes to the hot debate over why animals help others at their own expense. Theory suggests that helpers might benefit through increasing their own genetic success by helping relatives, or obtain future benefits such as inheriting the territory (pay-to-stay). Disentangling these alternatives is difficult, but anemonefishes provide a rare case where kinship is absent, which is why we could show that pay-to-stay is a viable option.

## Introduction

The mechanisms that select for the evolution of helping behavior in cooperative breeders remain a hotly debated topic (Clutton-Brock 2002; Lehmann and Keller 2006; Downing et al. 2020; Kay et al. 2020). The earliest and most extensively studied mechanism is outlined by “kin selection theory”, which proposes that individuals help close genetic relatives to reproduce, thereby indirectly promoting the proliferation of their own genes (Hamilton 1963; West-Eberhard 1975; Downing et al. 2020; Kay et al. 2020). Kin selection alone, however, cannot fully explain the evolution of helping, as some helpers are unrelated to the group members they are benefitting (Taborsky and Limberger 1981; Dunn et al. 1995; Queller et al. 2000). Alternative hypotheses have therefore been proposed to explain the evolution of helping behavior via direct benefits to helpers, such as acquiring parentage (Cockburn 1998), gaining skills (Skutch 1961), signaling quality to future partners (Zahavi 1995), enhancing group size (Kokko et al. 2001) or future territory inheritance (Kokko et al. 2002). We now know from several taxa that multiple interacting mechanisms select for the evolution of helping across cooperative breeders (Clutton-Brock 2002; Kingma et al. 2011; Taborsky 2016; Griesser et al. 2017; Koenig 2017; Cousseau et al. 2022; García-Ruiz et al. 2022). While theoretical models and long-term field studies provide insight into the relative influence of these mechanisms (Jungwirth and Taborsky 2015; Quinones et al. 2016; García-Ruiz et al. 2022), empirical evidence that direct benefits alone can drive the evolution of helping is lacking.

It remains challenging to empirically separate the effects of direct and indirect fitness benefits of helping, particularly in natural populations where relatedness, group size and social hierarchies are often intertwined (West et al. 2021). In most cooperatively breeding species, delayed dispersal of offspring results in the formation of groups composed of close relatives (Emlen 1995; Ekman et al. 2004; Heg et al. 2008; Clutton-Brock and Lukas 2012). In several taxa, helpers will start off by helping closely related breeders and then continue to help after their relatives are replaced with unrelated breeders (Taborsky and Limberger 1981; Dunn et al. 1995; Dierkes et al. 2005). This may suggest that whilst direct benefits are important in the expansion of helping to nonrelatives, kin selection, conferring indirect benefits, may be crucial to the initial evolution of helping. In support of this, many cooperative social groups of nonrelatives appear to have evolved from lineages of kin-based groups (Ekman and Ericson 2006; Berg et al. 2012; Riehl 2013). Further investigation is therefore required to determine whether helping behavior can evolve without the influence of kin selection.

Marine fishes, such as anemonefishes (*Amphiprion spp.*) may provide insight into whether helping behavior can evolve in the absence of kin selection. Many aspects of the social organization in anemonefishes resemble those of cooperative breeding species, with stable groups that maintain a strict divide between dominant breeders and subordinate nonbreeders (Fricke 1979; Buston 2004b; Rueger et al. 2018; Roux et al. 2020), however there are several key differences (Rueger et al. 2021): (i) anemonefishes, like nearly all marine animals, have a dispersive larval phase where larvae enter the water column upon hatching (Shanks 2009), and while juveniles sometimes make their way back to natal reefs (termed “self-recruitment”) (Jones et al. 1999), there is no delayed dispersal that would allow for group augmentation effects; (ii) relatedness within groups is no higher than in the population (Buston et al. 2007; Rueger et al. 2025a), excluding kin selection as an explanation for helping behaviors; (iii) nonbreeders do not directly care for the young of the group (Buston 2004a). However, while direct alloparental care is primarily absent, aside from occasional observations of defence against egg predators, nonbreeders perform other helping behaviors, such as territory maintenance and defence, presumably benefiting the group through improved territory quality and survival (Rueger et al. 2022; Yllan et al. 2024). While the fitness benefits of these behaviors remain unquantified, we know that behavioral interactions with the anemone elevates oxygen uptake, and anemone growth is influenced by both group size and fish behavior (Holbrook et al. 2005; Szczebak et al. 2013; Schmiege et al. 2017). Maintaining and protecting the anemone is likely to provide long-term fitness benefits to dominant anemonefish, as reproductive success is positively related to female size, which in turn depends on anemone size (Buston 2002; Buston and Elith 2011). Territory defence against food competitors and predatory fish may further enhance fitness by increasing food availability and survival. We therefore classify these behaviors as helping, based on the assumption that they confer fitness benefits to dominants and following definitions of helping as any action that provides a benefit to recipients (Taborsky et al. 2021), including both altruistic and mutually beneficial behaviors (Gardner et al. 2009). While these behaviors may appear more directly self-serving than alloparental care in cooperative breeders, both involve direct benefits to the actor, and they are likely to evolve and be maintained through similar mechanisms. Studying why unrelated subordinates in anemonefish groups perform these behaviors allows for investigating helping behavior in the complete absence of indirect fitness benefits.

Here, we investigate “pay-to-stay” as a mechanism selecting for helping behavior in anemonefishes. The pay-to-stay hypothesis proposes that nonbreeding individuals help dominant breeders to avoid eviction from the group, thereby gaining survival benefits such as protection from predators, and with the potential of gaining breeding positions in the future (Gaston 1978; Kokko et al. 2002). Pay-to-stay has proved a useful framework to enhance our understanding of helping across cooperatively breeding species, including mammals (Rood 1990; Reeve 1992), birds (Russell and Rowley 1988; Kingma 2017), freshwater cichlids (Taborsky 1985; Balshine-Earn et al. 1998) and insects (Reeve and Gamboa 1983; Field et al. 1999; Leadbeater et al. 2011). According to the pay-to-stay hypothesis, nonbreeders will be punished if not helping sufficiently and/or should increase their cooperative effort thereafter to appease the dominants (Bergmüller and Taborsky 2005). For example, in cichlids, subordinates who fail to participate in territory defence are punished or they increase their defence effort (Naef and Taborsky 2020a, 2020b), and punishment can lead to an increase in helping effort (Hidaka et al. 2024). However, the evolution of helping in cichlids is shaped by a combination of pay-to-stay (Taborsky 1985; Balshine-Earn et al. 1998), kin selection (Wong and Balshine 2011; Garcia-Ruiz and Taborsky 2024), and group augmentation (Garcia-Ruiz and Taborsky 2022), making it challenging to assess the relative contribution of pay-to-stay over alternative hypotheses for the evolution of helping behavior. In anemonefishes, the context for why subordinates accept their nonbreeding position is in line with pay-to-stay assumptions: (i) there are harsh ecological constraints preventing them from seeking alternative breeding habitats (Buston 2003a; Branconi et al. 2020); (ii) there are harsh social constraints preventing them from contesting for a breeding position (Buston 2003b; Rueger et al. 2018); (iii) subordinates stand to inherit the anemone territory (Buston 2004b). Additionally, the assumptions of the main alternative hypothesis explaining helping are not met in anemonefishes (Table 1), allowing exploration of pay-to-stay mechanisms independently of alternative direct benefits.

**Table 1:** The main hypotheses explaining the evolution of helping in cooperative breeders, highlighting the assumptions of each hypothesis and whether these are met in anemonefishes (*Amphiprion spp.*).

Hypothesis	Concept	Key assumptions	Assumptions met in *Amphiprion spp*.?
Kin selection (Hamilton 1963)	Helpers indirectly promote proliferation of their own genes.	Helping is directed towards relatives.	**No**: Relatedness within groups is not higher than in the population (Buston et al. 2007; Rueger et al. 2025a).
Parentage acquisition (Cockburn 1998)	Helpers obtain access to breeding opportunities.	Helpers acquire parentage.	**No**: Reproductive suppression in helpers (Rueger et al. 2018).
Skills acquisition (Skutch 1961)	Helpers acquire experience, improving their success as breeders in the future.	Individuals with helping experience have higher success as breeders. Helping effort decreases with age and experience.	Untested **No**: Higher ranked subordinates (older, more experienced) help more (Rueger et al. 2022).
Social prestige (Zahavi 1995)	Helpers signal quality to potential future partners.	Helpers inherit breeding position in future. Same-sex competition and unstable queues.	**Yes**: Subordinates inherit territory following death of dominant (Buston 2004b). **No**: Strict size hierarchies ensure stable queues (Buston 2003).
Group augmentation (Kokko et al. 2001)	Helpers benefit from larger group sizes by enhancing breeder productivity.	Living in larger groups is beneficial. Individuals help more in smaller groups. Offspring delay dispersal and are recruited to join group	Untested **No:** Group size does not affect helping effort (Rueger et al. 2022). **No**: Larvae disperse immediately after hatching (Shanks 2009).
Pay-to-stay (Kokko et al. 2002)	Helpers appease dominant breeders to avoid eviction from the territory.	Helpers gain benefits from staying in the group, such as reduced predation. Punishment and/or pre-emptive appeasement following insufficient helping.	**Yes**: Subordinates do not move to unoccupied anemones due to high mortality risk outside of territory (Branconi et al. 2020). **Yes:** The current study reveals that punishment and/or pre-emptive appeasement occurs following prevention of helping

Adapted from Kingma et al. (2011) and Cousseau et al. (2022).

We explored pay-to-stay as a driver of helping behavior in 3 anemonefish species: *A. percula*, *A. perideraion*, and *A. clarkii*. Anemonefish social groups typically consist of a single breeding pair and several nonbreeding subordinates (Buston 2004b; Fricke 1979). These groups form strict size hierarchies; the dominant female is the largest, followed by the male, with subordinates decreasing in size down the hierarchy. Species show subtle differences in their ecological constraints and social behaviors that likely alter the pay-offs associated with paying to stay. *Amphiprion percula* live in the smallest groups, usually between 2 and 6 individuals, and do not leave their host anemone following settlement due to harsh ecological constraints (Buston 2002; Branconi et al. 2020). *Amphiprion perideraion* live in groups of up to 12 individuals, are more aggressive and less cooperative than *A. percula*, and large individuals can sometimes leave their host anemones (Rueger et al. 2022, 2025b). In *Amphiprion clarkii*, group size can be as large as 15 and ecological constraints are relaxed with movement between groups more frequent (Hattori 2002; Cleveland et al. 2011). Studying pay-to-stay mechanisms in multiple species allows us to evaluate how social structure and ecological constraints shape these dynamics.

To investigate, we first established how baseline behavior differs between species. We then used a field experiment, in which subordinates were prevented from helping to test the 2 key predictions of the pay-to-stay hypothesis: (i) Subordinates will receive increased aggression from dominants after being prevented from helping—punishment; (ii) Subordinates will increase their cooperative effort, via helping and/or proactive submission, following prevention of helping—pre-emptive appeasement. Additionally, as growth suppression in subordinate anemonefish has been equated as a form of “rent payment” (Buston 2003b, 2004a; Buston and Cant 2006; Wong et al. 2007), we also explore whether pay-to-stay dynamics are influenced by the relative size of subordinates, compared with their dominants.

## Materials and methods

### Study site and experimental groups

This study was carried out on inshore reefs near Mahonia Da Nari Research and Conservation Center, in Kimbe Bay, Papua New Guinea (5°30′ S, 150°05′ E) from February to April 2023. All observations, experimental manipulations and measurements were carried out in situ via SCUBA. The sample sizes used in analysis were 12 groups of *A. percula*, 12 groups of *A. perideraion* and 6 groups of *A. clarkii*. Both *A. percula* and *A. perideraion* groups were comprised of either 3 (*n* = 10 and 9, respectively) or 4 (*n* = 2 and 3, respectively) individuals and *A. clarkii* groups were comprised of 3 (*n* = 3), 4 (*n* = 2) or 5 (*n* = 1) individuals. All *A. percula* and *A. perideraion* groups were associated with the anemone species *Radianthus magnifica* (previously known as *Heteractis magnifica*) (Titus et al. 2024), which both species primarily associate with in the region. For *A. clarkii*, most groups (*n* = 4) were associated with *Stichodactyla mertensii* and some (*n* = 2) were associated with *Heteractis crispa*.

To exclude behavioral differences due to breeding, only nonbreeding groups were selected. When breeding was observed during the experiment, we concluded the experiment and excluded these groups from analyses. We used GPS coordinates to locate reefs and numbered identification tags (attached to substrate near the anemone) to locate each group, which allowed for repeated measures. At least 1 day prior to any observations or experimental manipulations, we measured both anemone size and each group members' size. We used tailor's tape to measure the long and short diameter of the anemone tentacle crown to approximate surface area (π × major axis/2 × minor axis/2). We caught all individuals using hand nets, transferred each individual into a clear ziplock bag and measured their standard-length (SL) using calipers. This allowed us to account for any effects of anemone size, group size and between-individual size ratios on behavior.

### Experimental protocol

**Figure 1:**
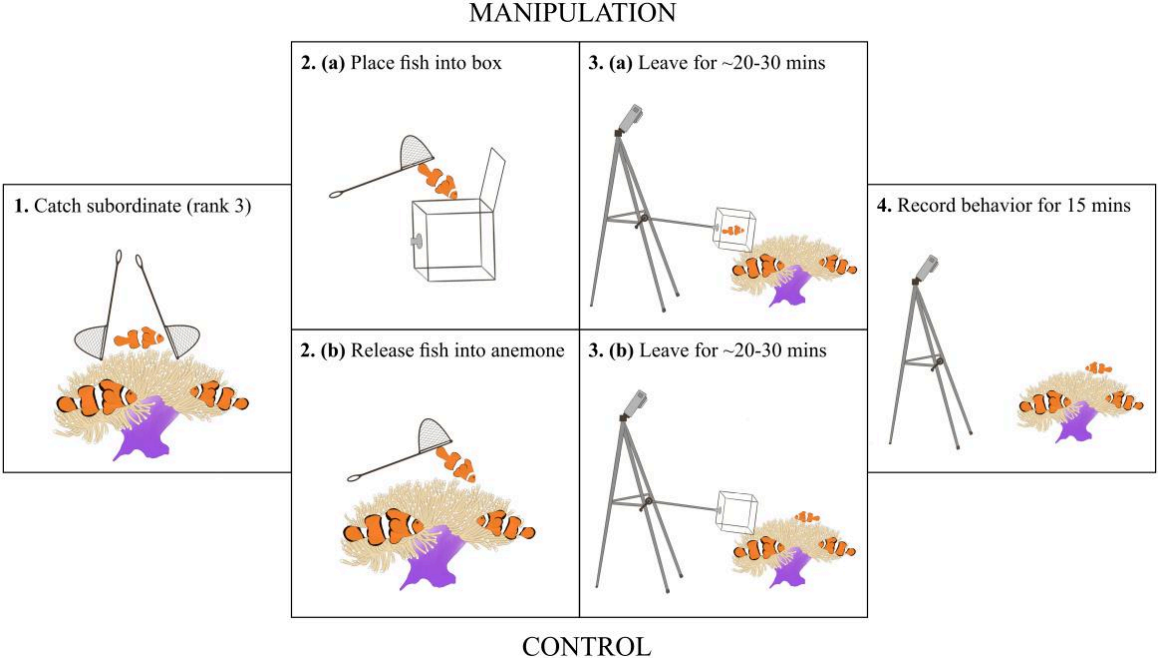
Experimental design of helping prevention experiment. Repeated measures design conducted with groups of *A. percula* (*N* = 12), *A. perideraion* (*N* = 12) and *A. clarkii* (*N* = 6). In the manipulation treatment a), the focal subordinate was placed in a clear box to prevent helping. The control treatment b) controlled for the effects of catching and presence of the box. Baseline behavioral recordings were carried out using the same set up as in control and treatment conditions (step 4), but without the preceding steps.

We experimentally prevented helping behaviors in the highest-ranked subordinate from each group to investigate the key predictions of pay-to-stay (punishment and pre-emptive appeasement following insufficient helping). The experiment had a repeated-measures design with a treatment (ie, helping prevention) and control condition (Fig. 1). All groups were tested once in each condition over consecutive days, with the order counterbalanced to account for habituation to the equipment. In the treatment condition, we caught the highest-ranking subordinate (ie, rank 3) using hand nets and placed them in a clear plastic box (15 × 15 × 15 cm) for approximately 25 min (between 20 and 33 min). After this period, the focal subordinate was released back into their original anemone host, the box was removed and behavioral recordings followed. The control condition aimed to account for both the effects of catching the subordinate and the presence of the equipment. The protocol for the control was therefore identical, except the subordinates were immediately released back to their anemone host rather than being placed in the box. The boxes were positioned as close to the anemone as possible but without interfering with the anemone tentacles (∼10 to 20 cm from anemone tentacles). This aimed to limit stress to the focal fish from being distanced from its host and ensure their presence was observed by other group members. The boxes were fitted with holes to allow for oxygen flow and transfer of chemical cues that may signal identity. The focal subordinate's perceived presence in the group was crucial, as temporary removal of helpers has been shown to produce different responses to confinement (Fischer et al. 2014), and we aimed to minimize disruption to the social structure impacting our results (Naef and Taborsky 2020a). The perceived presence of the focal subordinate was confirmed by observations of other group members approaching the fish within the box.

### Behavioral recordings and analysis

All groups were filmed on 4 occasions: 2 pretreatment recordings (day 1 and 2) to measure baseline behavior and 2 post-treatment recordings, 1 following each experimental condition (day 3 and 4). Filming was carried out without divers present with a GoPro Hero 5 from a distance of 1.5 to 2.5 m for 17 min (step 4, Fig. 1). To exclude the influence of diver disturbance during equipment set up, we removed 1 min from the start and end of each 17 min recording, resulting in a 15 min observation period. This protocol is consistent with previous studies recording behaviors of anemonefishes in the wild (Wong et al. 2017; Rueger et al. 2022; Yllan et al. 2024).

Behaviors were scored from videos using the BORIS programme (Friard and Gamba 2016), following an ethogram based on (Rueger et al. 2022) (Supplementary material S1). We scored the following behaviors for the focal subordinate: (i) aggression received from dominants, (ii) proactive submission (submissive behaviors not directly in response to received aggression) towards dominants, and (iii) “helping” behaviors (anemone maintenance, defence against predators and food competitors). We created the novel behavioral category “proactive submission” to distinguish pre-emptive appeasement from defensive submission. Proactive submission included both avoidance behavior and submissive displays. For both aggression and proactive submission, we recorded whether it was rank 1 (male) or rank 2 (female) that displayed or received the behavior, respectively. Individuals were identified from videos by their unique body patterns and size differences. All behaviors were recorded as state events (ie, durations in seconds). To account for differences in the times that fish were visible, we recorded out of sight times. We then calculated the duration of each behavior and expressed it as a proportion of the total time the focal subordinate was visible during the observation period.

Social interactions were scored by 1 observer and helping behaviors and out of sight times were scored by another observer. Both observers scored each observation continuously in 1 sequence. Prior to scoring, observers watched multiple videos together to agree on how to score the behaviors. Because the boxes were visible in day 3 and 4 videos, observers were not blind to the treatment. However, treatments were scored in an arbitrary order to avoid unconscious patterning of the data.

### Data analysis

All analyses were conducted using R version R 4.5.1 (R Core Team 2024). We employed a Bayesian modeling approach to support stable estimation of complex models (particularly zero-inflation models), for improved performance at relatively small sample sizes and to allow direct quantification of parameter uncertainty. All models were fitted using the *brms* package (Bürkner 2017, 2018) via *cmdstanr* (Gabry et al. 2025), specifying beta or zero-inflated beta likelihoods as appropriate. Because the beta distribution cannot accommodate zeros, we added a small constant of 0.001 to all values of the response variable in datasets containing 1 or 2 zeros. For datasets with a higher number of zeros (10 to 56% of observations), we employed zero-inflated models.

All models (across species and behavioral responses) were fitted using the same weakly informative prior structure, following prior predictive checks (Supplementary material Figure S1). Intercept priors were specified to ensure behavioral responses remained biologically plausible (generally <25% of observation time). Continuous predictors were *z*-standardized (mean = 0, SD = 1) prior to analysis. Regression coefficients (β) were assigned nondirectional Normal (0, 1) priors. Candidate models were compared with leave-one-out cross-validation (LOO) using *loo* package (Vehtari et al. 2017), evaluating performance using differences in expected log predictive density (elpd_diff) and associated standard errors. Candidate predictors considered of primary biological interest or previously shown to influence behavior were retained unless their inclusion substantially reduced predictive accuracy (elpd_diff > 2 × SE). In contrast, additional covariates were included only when they improved predictive performance (elpd_diff ≥ 1 × SE). This approach prioritizes model predictive accuracy while allowing biologically motivated effects to be retained when performance is not compromised.

To explore behavioral differences between species and ranks, we fitted separate models for each broad behavioral category: aggression, proactive submission and helping. We used mean baseline behaviors (ie, average across 2 pretreatment recordings) as response variables. All models included species (*A. percula, A. perideraion, A. clarkii*) as a fixed factor. The size ratio between the focal rank 3 and its immediate dominant (rank 3 SL/rank 2 SL), a biologically meaningful predictor, was retained in final models for proactive submission and helping as its inclusion did not reduce predictive accuracy (elpd_diff ≥ 2 × SE). Additional covariates (group size and anemone size) were excluded from all final models because they did not improve predictive performance. For aggression and proactive submission, rank (R1, R2) was included as a fixed factor and group ID was treated as a random variable to account for the nonindependence of individual behaviors towards or from each rank. An interaction between species and rank, considered of primary interest to explore rank effects within species, was retained in both final models as it did not reduce predictive accuracy (elpd_diff ≥ 2 × SE).

To address whether punishment and appeasement occurred following helping prevention, as predicted by pay-to-stay, we first calculated the total proportion of time that aggression was received and proactive submission was performed by both ranks. We fitted separate models for each specific behavioral category (aggression, proactive avoidance, proactive displays, anemone maintenance, territory defence) and each species (*A. percula, A. perideraion, A. clarkii*). All models included treatment (baseline, control, manipulation) as a fixed factor and group ID as a random factor to account for repeated measures of the same individuals. For *A. clarkii*, given the small sample size (*n* = 6), we did not perform model selection; instead, simple models including only treatment and group ID were used to avoid overfitting. In *A. percula* and *A. perideraion* models, an interaction between size ratio and treatment was retained in all final models as its inclusion did not reduce predictive accuracy (elpd_diff ≥ 2 × SE). This allows us to examine the trade-offs between paying by remaining small and paying through cooperative effort. Additional covariates (group size and anemone size) were excluded from all final models because they did not improve predictive performance.

All final models were screened for convergence, adequate effective sample sizes (bulk and tail ESS > 1000), suitable model shrinking (R̂ values <1.05) and autocorrelation (adequate mixing of Markov chains in ACF plot). Predictive performance was validated with LOO (Pareto *k* values <0.7) and posterior predictive checks assessed model fit by comparing observed data to simulated datasets. To explore behavioral differences between species, ranks and treatments, post-hoc pairwise comparisons were carried out. Posterior predicted values were generated from models using the *tidybayes* package (Kay 2024a) and pairwise contrasts were computed from posterior draws using the *marginaleffects* package (Arel-Bundock et al. 2024). To evaluate the treatment × size ratio interaction, we defined the categories of closer in size (size.ratio_*z* > 0) and further in size (size.ratio_*z* < 0) to immediate dominants (where size.ratio_*z* is the *z*-standardized size ratio). Posterior predictions were generated across the observed range of size.ratio_*z* and contrasts between treatment were computed within each size ratio category. The main effect of size ratio was quantified by extracting posterior draws using the *posterior* package (Bürkner et al. 2025). Posterior predictions were summarized using medians and 89% highest density intervals (HDIs) to characterize effect sizes and associated uncertainty. Following McElreath (2020), 89% intervals were used as a stable summary of the central posterior distribution, while 95% HDIs are additionally shown in figures to visualize uncertainty across wider intervals. Figures present posterior predictions on the response scale (percentage of time spent displaying the behavior) to facilitate direct interpretation. Predicted percentage differences relative to reference group are also reported to quantify relative differences in magnitude between groups. Directional evidence was quantified as the posterior probability that an effect exceeded zero (*P*(θ > 0)). Model visualization was conducted using the *ggplot2* (Wickham et al. 2016) and *ggdist* (Kay 2024b) packages, with color palettes from the *colorspace* package (Zeileis et al. 2020). Figures were exported from R and refined using Affinity Designer.

### Ethical note

This study was conducted with the approval of Newcastle University's Animal Welfare Ethical Review Body (AWERB; Project ID: 1030) and the Government of Papua New Guinea. All work conducted adhered to the ABS/ASAB guidelines for ethical treatment of animals.

## Results

### Species and rank behavioral differences

**Figure 2:**
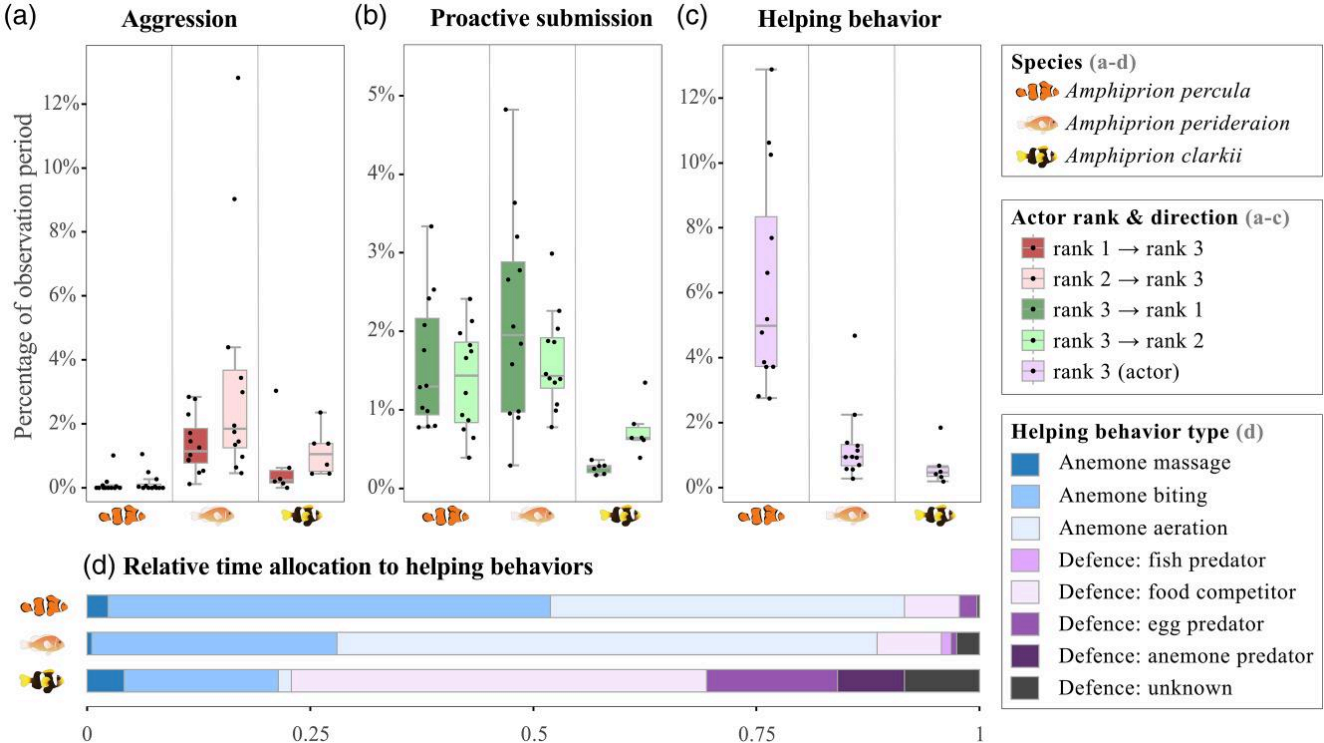
Species comparison of baseline behaviors (as a percentage of the total time visible) for *A. percula* (*N* = 12), *A. perideraion* (*N* = 12) and *A. clarkii* (*N* = 6). Central bar: median; boxes: interquartile range (IQR); whiskers: ± 1.5×IQR; dots: observed values. a) Aggression received, b) proactive submission, and c) helping behavior (anemone maintenance and territory defence) are shown by actor rank (and direction). d) Relative time allocation to each helping behavior, with defence categorized by intruder type; no anemone cleaning was observed.

Baseline behavioral patterns differed between species and ranks (Fig. 2; Supplementary material S2–S4). Posterior contrasts indicate that *A. perideraion* were more aggressive than *A. clarkii* (median Δ = 0.0102, 89% HDI [0.0027, 0.0178], Pr(Δ > 0) = 0.983) and *A. percula* (median Δ = 0.0159, 89% HDI [0.0097, 0.0220], Pr(Δ > 0) > 0.999). Although absolute differences (ie, median Δ) may appear small, these correspond to approximately 101% and 350% higher predicted aggression relative to *A. clarkii* and *A. percula*, respectively. There was also evidence that *A. clarkii* were more aggressive than *A. percula* (median Δ = 0.0057, 89% HDI [0.0006, 0.0111], Pr(Δ > 0) = 0.970), corresponding to ∼55% lower aggression in *A. percula*. Within species, dominant males (rank 2) performed more aggression than females (rank 1) in *A. perideraion* (median Δ = 0.0110, 89% HDI [0.0017, 0.0203], Pr(Δ > 0) = 0.973). Evidence for the same pattern was weaker in *A. percula* (Pr(Δ > 0) = 0.827) and *A. clarkii* (Pr(Δ > 0) = 0.863).

Proactive submission was less common in *A. clarkii* compared with *A. percula* (median Δ = 0.0083, 89% HDI [0.0043, 0.0125], Pr(Δ > 0) = 0.999) and *A. perideraion* (median Δ = 0.0109, 89% HDI [0.0063, 0.0156], Pr(Δ > 0) > 0.999), corresponding to approximately 113% and 147% higher submission in *A. percula* and *A. perideraion*, respectively. There was weaker evidence that *A. perideraion* were more submissive than *A. percula* (Pr(Δ > 0) = 0.796). Within species, we found no clear evidence for differences in submission towards rank 1 versus rank 2 in *A. percula* (Pr(Δ > 0) = 0.439) and *A. perideraion* (Pr(Δ > 0) = 0.317) and only weak evidence indicating greater submission toward rank 2 individuals in *A. clarkii* (Pr(Δ > 0) = 0.797).

Helping behaviors (anemone maintenance and defence) were most frequent in *A. percula*. Posterior contrasts indicate that *A. percula* displayed more helping than both *A. perideraion* (median Δ = 0.0423, 89% HDI [0.0298, 0.0522], Pr(Δ > 0) > 0.999) and *A. clarkii* (median Δ = 0.0454, 89% HDI [0.0342, 0.0589], Pr(Δ > 0) = 0.999), which corresponds to 391% more helping in *A. percula* relative to *A. clarkii*, and a 74% less helping in *A. perideraion* relative to *A. percula*. There was weaker evidence that *A. perideraion* perform more helping behaviors than *A. clarkii* (Pr(Δ > 0) = 0.764). Across species, subordinates closer in size to their immediate dominant spent more time performing helping behaviors (median Δ = 0.2078, 89% HDI [0.0208, 0.3764], Pr(Δ > 0) = 0.934).

### Punishment following helping prevention

**Figure 3:**
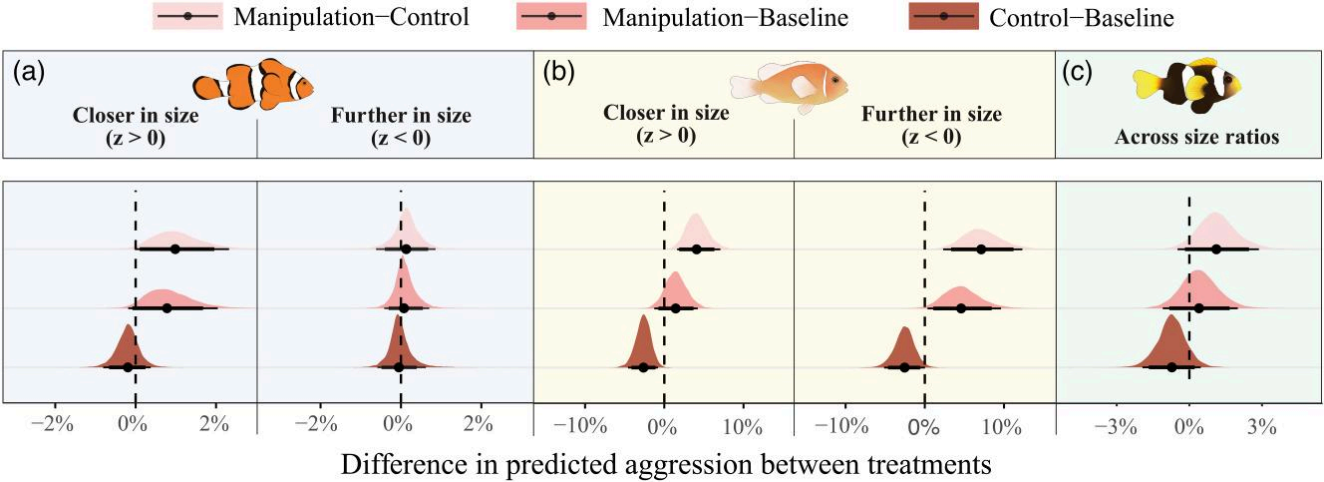
Posterior contrasts in aggressive behavior between conditions (baseline, control, manipulation) for a) *A. percula* (*N* = 12), b) *A. perideraion* (*N* = 12), and c) *A. clarkii* (*N* = 6). For *A. percula* and *A. perideraion*, contrasts are shown for individuals closer in size (*z* > 0) and further in size (*z* < 0) from their immediate dominant. The dashed vertical line at zero indicates no difference between conditions. The points display predicted medians and horizontal bars represent 89% and 95% HDIs.

The experimental prevention of helping led to more aggression towards the focal subordinate in 2 of the 3 species—*A. percula* and *A. perideraion* (Fig. 3; Supplementary material S5–S7). In *A. percula*, the effect of treatment depended on size ratio; when the focal subordinate was close in size to their immediate dominant (*z* > 0), posterior contrasts indicate greater aggression following the helping prevention, compared with both baseline (median Δ = 0.0078, 89% HDI [−0.0004, 0.0168], Pr(Δ > 0) = 0.957) and control conditions (median Δ = 0.0098, 89% HDI [0.0010, 0.0196], Pr(Δ > 0) = 0.981), which corresponds to ∼100% and 170% increase, respectively. Whereas there was no clear evidence for an effect of treatment at smaller size ratios (*z* < 0). In *A. perideraion*, aggression was also affected by treatment, with only weak evidence for an effect of size ratio, though the magnitude of increased aggression tended to be higher at larger size differences. We found greater aggression following the helping prevention, compared with both baseline (median Δ = 0.0361, 89% HDI [−0.0083, 0.0650], Pr(Δ > 0) = 0.989) and control (median Δ = 0.0616, 89% HDI [0.0326, 0.0920], Pr(Δ > 0) > 0.999) conditions, corresponding to an increase of ∼78% and 304%, respectively. Aggression in the control condition was also lower compared with baseline (median Δ = −0.0257, 89% HDI [−0.0421, −0.0106], Pr(Δ > 0) = 0.004). In *A. clarkii*, we found limited evidence for an increase in aggression following helping prevention. Whilst there was some evidence that aggression was higher following the helping prevention compared with the control condition (median Δ = 0.0111, 89% HDI [−0.0018, 0.0246], Pr(Δ > 0) = 0.932), evidence for a difference between manipulation and baseline conditions was limited (Pr(Δ > 0) = 0.713), and in both cases HDIs indicate uncertainty.

### Appeasement via proactive submission following helping prevention

**Figure 4:**
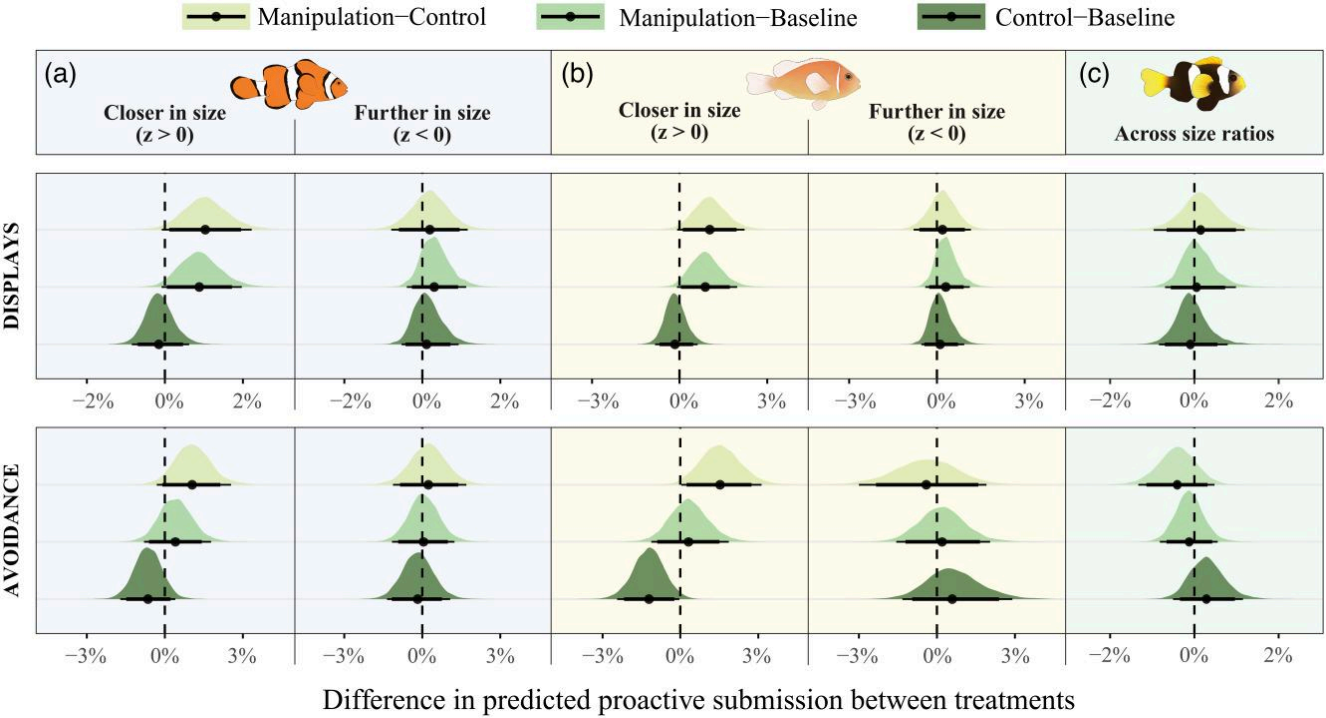
Posterior contrasts in proactive submission (displays and avoidance) between conditions (baseline, control, manipulation) for a) *A. percula* (*N* = 12), b) *A. perideraion* (*N* = 12), and c) *A. clarkii* (*N* = 6). For *A. percula* and *A. perideraion*, contrasts are shown for individuals closer in size (*z* > 0) and further in size (*z* < 0). The dashed vertical line at zero indicates no difference between conditions. The points display predicted medians and horizontal bars represent 89% and 95% HDIs.

The experimental prevention of helping led to more proactive submission by the focal subordinate in the same 2 species—*A. percula* and *A. perideraion* (Fig. 4; Supplementary material S8–S13). In both species, the effect of treatment depended on size ratio. When the focal subordinate was close in size to their immediate dominant (*z* > 0), posterior contrasts indicated more submissive displays following the helping prevention, compared with both baseline (*A. percula*: median Δ = 0.0088, 89% HDI [0.0005, 0.0172], Pr(Δ > 0) = 0.969; *A. perideraion*: median Δ = 0.0137, 89% HDI [0.0044, 0.0237], Pr(Δ > 0) = 0.992) and control conditions (*A. percula*: median Δ = 0.0103, 89% HDI [0.0011, 0.0195], Pr(Δ > 0) = 0.970; *A. perideraion*: median Δ = 0.0198, 95% HDI [0.0096, 0.0297], Pr(Δ > 0) = 0.999). This corresponds approximately to a 95% and 88% increase relative to baseline, and 134% and 212% increase relative to control for *A. percula* and *A. perideraion*, respectively. Submissive displays (when size ratio *z* > 0) were also less common in the control condition compared with baseline in *A. perideraion* (median Δ = −0.0061, 89% HDI [−0.0116, −0.0005], Pr(Δ > 0) = 0.046).

There was limited evidence for an effect of treatment on proactive avoidance behavior. Whilst posterior contrasts indicate increased avoidance (when size ratio *z* > 0) following the helping prevention compared with the control condition in *A. perideraion* (median Δ = 0.0153, 89% HDI [0.0024, 0.0274], Pr(Δ > 0) = 0.984), and slightly weaker evidence for a similar pattern in *A. percula* (median Δ = 0.0105, 89% HDI [−0.0009, 0.0251], Pr(Δ > 0) = 0.943), there is limited evidence to suggest an increase in avoidance relative to baseline in both species (*A. percula*: Pr(Δ > 0) = 0.742; *A. perideraion*: Pr(Δ > 0) = 0.670). There was also no clear evidence for an effect of treatment in *A. clarkii* for both submissive displays and avoidance behavior.

### Appeasement via helping following helping prevention

**Figure 5:**
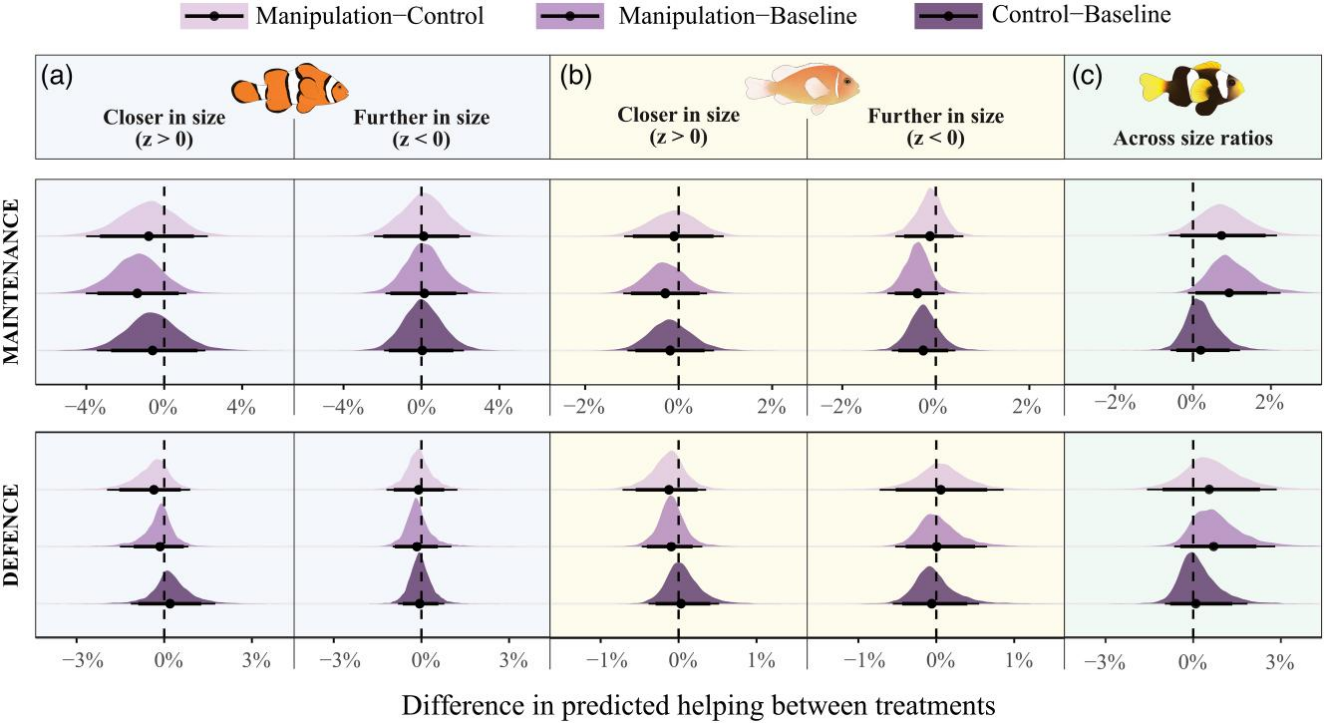
Posterior contrasts in helping behavior (anemone maintenance, territory defence) between conditions (baseline, control, manipulation) for a) *A. percula* (*N* = 12), b) *A. perideraion* (*N* = 12), and c) *A. clarkii* (*N* = 6). For *A. percula* and *A. perideraion*, contrasts are shown for individuals closer in size (*z* > 0) and further in size (*z* < 0). The dashed vertical line at zero indicates no difference between conditions. The points display predicted medians and horizontal bars represent 89% and 95% HDIs.

The experimental prevention of helping led to an increase in helping behavior, specifically anemone maintenance, by the focal subordinate in 1 of the 3 species—*A. clarkii* (Fig. 5, Supplementary material S14–S19). In this species, posterior contrasts indicate an increase in anemone maintenance following helping prevention compared with baseline (median Δ = 0.0093, 89% HDI [0.0006, 0.0191], Pr(Δ > 0) = 0.976) and control conditions (median Δ = 0.0073, 89% HDI [−0.0033, 0.0187], Pr(Δ > 0) = 0.877), which corresponds to ∼166% and 96% increase, respectively (although the HDI indicates some uncertainty in the control contrast). There was no clear evidence of a treatment effect on territory defence behaviors in any of the 3 species, nor on anemone maintenance in *A. percula* and *A. perideraion*. However, anemone maintenance was more frequent in subordinates closer in size to their immediate dominants in *A. percula* (Pr(Δ > 0) = 0.976), and there was weaker evidence for the same pattern in *A. perideraion* (Pr(Δ > 0) = 0.9346).

## Discussion

We found that pay-to-stay mechanisms can drive helping behavior in the absence of kin selection. In all 3 anemonefish species investigated, punishment and/or pre-emptive appeasement occurred in response to the prevention of helping, as predicted by the pay-to-stay hypothesis (Kokko et al. 2002; Bergmüller and Taborsky 2005). The helping prevention elicited varied responses. In 2 out of the 3 species, subordinates received higher levels of aggression, indicating punishment for insufficient helping effort or lack of appeasement. We observed 2 distinct forms of pre-emptive appeasement: increased proactive submission displays and increased helping behavior, specifically anemone maintenance. These results indicate that diverse behavioral strategies emerge to mitigate conflict in closely related species, depending on ecological and social constraints. Interestingly, in 2 species, punishment and pre-emptive appeasement varied according to the size ratio between subordinates and their immediate dominant, indicating a trade-off between growth and cooperative effort.

By investigating pay-to-stay in a social system where delayed dispersal is absent and relatedness within groups is not elevated (Jones et al. 1999; Buston et al. 2007; Rueger et al. 2025a)—thus excluding indirect genetic benefits—we demonstrate that helping behaviors can evolve independently of kin selection. Kin selection may therefore not be as central to the evolution of helping as is often argued (Hamilton 1963; West-Eberhard 1975; Foster et al. 2006; Downing et al 2020; Kay et al. 2020) and more tests on species without delayed dispersal are necessary. Previous research has shown that subordinate anemonefishes accept their nonbreeding situation, despite the lack of indirect genetic benefits, due to harsh ecological and social constraints and the future prospect of territory inheritance (Rueger et al. 2021; Buston 2004b). Building on these findings, our study provides insight into why subordinates might engage in anemone maintenance and defence behaviors and why dominant breeding pairs tolerate other group members in their territory. To provide further support, future work should aim to test additional coercion dynamics underlying pay-to-stay, specifically demonstrating that increased cooperation leads to greater tolerance by dominants and that punishment promotes increased cooperation. Subordinate anemonefishes are unlikely to help for reasons related to parentage, skill acquisition, quality signaling, or group augmentation (Table 1), and thus coercion alone may effectively promote helping behavior. We suspect that coercion is enough to promote helping in anemonefishes due to the stringent ecological constraints, making breeding elsewhere nearly impossible (Fautin 1991; Elliott et al. 1995; Buston 2003a, 2003b; Branconi et al. 2020). This supports the view that ecological constraints and the resulting direct benefits of group living are the primary forces shaping the evolution of helping behavior in cooperatively breeding animals (Dunn et al. 1995; Queller et al. 2000; Gardner and West 2004; Wilson 2005; Quinones et al. 2016; García-Ruiz et al. 2022).

Because stringent environmental constraints are common in coral reef fishes with complex social groups (Wong 2010; Branconi et al. 2020), it is possible that pay-to-stay dynamics are more widespread among marine fishes than currently appreciated. On the other hand, anemonefishes may exhibit unique traits that have facilitated the evolution of complex cooperative mechanisms. While group territorial defence is widespread among reef fishes due to intense interspecific competition (Clifton 1990; Forrester 2015; Eurich et al. 2018a, 2018b, 2018c), anemonefishes may require greater collaboration in territory maintenance due to the mutualistic relationship with the anemone host (Fautin 1991; Holbrook et al. 2005; Cleveland et al. 2011). The importance of this mutualistic relationship to social evolution in anemonefishes is supported by our findings of appeasement via anemone maintenance, rather than defence, in *A. clarkii*. Notably, the only other documented example of punishment to promote cooperation among marine fishes is found in cleaner wrasses (*Labroides dimidiatus*), where punishment reinforces mutualistic interactions with client fish (Bshary and Grutter 2005). Exploring the role of mutualisms and reciprocity in shaping the evolution of cooperation among marine fishes represents an intriguing avenue for future research.

The diverse pay-to-stay strategies observed among the 3 anemonefish species likely reflect differences in social structure and ecological constraints (Buston 2002; Cleveland et al 2011; Branconi et al. 2020; Rueger et al. 2022). In both *A. percula* and *A. perideraion*, subordinates were punished and engaged in pre-emptive appeasement through submissive displays. However, *A. perideraion* received more punishment and showed slightly higher levels of appeasement than *A. percula*. This pattern is consistent with expectations, given that *A. percula* is the more cooperative species (Fig. 2; Rueger et al. 2022), and further supports this distinction by highlighting how *A. percula* resolve conflict more efficiently than *A. perideraion*. Several factors may explain the lack of punishment observed in *A. clarkii*. We suspect that pre-emptive appeasement effectively eliminated the need for punishment (Balshine-Earn et al. 1998; Bergmüller and Taborsky 2005; Zöttl et al. 2013; Naef and Taborsky 2020a; Hidaka et al. 2024). Alternatively, their ability to switch groups may reduce the effectiveness of punishment (Hattori 2002; Bergmüller et al. 2005; Cleveland et al. 2011) and their greater mobility and larger group sizes may hinder ability to detect defection (Hattori 2002; Cleveland et al. 2011; Fischer et al. 2014). We also note that the smaller sample size for *A. clarkii* may have reduced our power to detect meaningful differences here. Nonetheless, these pay-to-stay strategies employed by different species are likely context-dependant, as shown in cichlids, where strategy varies with predation threat, demand for maintenance, and the behavior prevented (Heg and Taborsky 2010; Naef and Taborsky 2020a, 2020b). Applying similar experimental approaches that explore different contexts in anemonefishes would help clarify the relative importance of different helping behaviors across species and under different ecological conditions.

We demonstrate that size ratios are important to account for when studying pay-to-stay strategies. The effect of size ratio was most pronounced in *A. percula*, where both punishment and appeasement occurred only at larger size ratios (ie, when subordinates were closer in size to their immediate dominant). As growth suppression by subordinate anemonefishes has been equated to rent payment and is comparable in function to helping (Buston 2003a, 2003b, 2004a, 2004b; Buston and Cant 2006; Wong et al 2007), this points to a trade-off between growth and helping effort. In *A. perideraion*, appeasement also only occurred at larger size ratios, however punishment occurred across size ratios and tended to be lower at larger size ratios. These interspecific differences may be explained by variation in size ratio structure: size hierarchies are stricter and more consistent in *A. percula*, whereas they appear to be more variable in *A. perideraion* (T Rueger, *personal communication*). The higher levels of punishment and lower levels of appeasement for *A. perideraion* further in size from their immediate dominant may suggest that this variable size ratio structure is linked to levels of cooperation. Those closer in size to dominants may pay less in growth suppression due to their increased cooperative nature or due to having less aggressive dominants, which may also explain the lower punishment. Despite evidence of growth suppression and size-based behavioral differences in other pay-to-stay systems (Clutton-Brock et al. 2005; Hamilton et al. 2005; Field et al. 2006; Bruintjes and Taborsky 2008, 2011), to our knowledge, the influence of size ratios on punishment and appeasement has not been explored in other taxa. We advocate for future studies to explore this trade-off, incorporating long-term monitoring of growth, cooperation and social dynamics.

In conclusion, our results demonstrate that pay-to-stay mechanisms drive helping behavior in anemonefishes, independently of kin selection and other direct benefits. These findings challenge many existing views of social evolution, providing further support that kin selection is not a necessary requirement for helping behavior and cooperative negotiation strategies to evolve. We highlight the diverse pathways through which complex social structures can emerge, proposing that the threat of eviction posed by dominants can be sufficient in driving the evolution of helping when ecological and social constraints are high. We reveal that sophisticated cooperation mechanisms extend to marine fishes, underscoring the need to investigate both the prevalence of such cooperation and the role of mutualisms in the evolution of marine sociality. Overall, by broadening our investigation of helping behavior to include marine fishes, which differ from traditionally studied cooperative breeding societies, we gain valuable insights into social evolution.

## Supplementary Material

arag061_Supplementary_Data

## Data Availability

Analyses reported in this article can be reproduced using the data and code provided by Gaffney et al. (2026).
