# Supplementary material for: Pay-to-stay drives the evolution of helping independent of kin selection in anemonefish societies
Source: Behav Ecol. 2026 Jun 10;37(4):arag061. doi: 10.1093/beheco/arag061 (PMC13308537; doi:10.1093/beheco/arag061)
Supplement: arag061_Supplementary_Data [file arag061_supplementary_data.docx]

**Supplementary Material:**

Author 1, Author 2, Author 3. 2025 Pay-to-stay drives the evolution of helping independent of kin selection in anemonefish societies. *Behavioral Ecology*.

**Contents**

Glossary of model terms and summary statistics…………………..………....……….………………..2

Anemonefish behaviour ethogram (Table S1)……….………….………….……………….……….…3

Model prior predictive distributions (Figure S1)………. …………………….…………………..……4

Baseline aggression: species and rank comparison (Table S2, Figure S2)...………....…………..….…5

Baseline proactive submission: species and rank comparison (Table S3, Figure S3)...........… ……….6

Baseline helping behaviour: species comparison (Table S4, Figure S4)................................…………7

Treatment effect on aggression in *A. percula* (Table S5, Figure S5)................................……….......…8

Treatment effect on aggression in *A. perideraion* (Table S6, Figure S6).......................……….........…9

Treatment effect on aggression in *A. clarkii* (Table S7, Figure S7)...............................….……......…10

Treatment effect on proactive submissive displays in *A. percula* (Table S8, Figure S8).....................11

Treatment effect on proactive submissive displays in *A. perideraion* (Table S9, Figure S9)………...12

Treatment effect on proactive submissive displays in *A. clarkii* (Table S10, Figure S10)....................13

Treatment effect on proactive submissive avoidance in *A. percula* (Table S11, Figure S11)...............14

Treatment effect on proactive submissive avoidance in *A. perideraion* (Table S12, Figure S12)……15

Treatment effect on proactive submissive avoidance in *A. clarkii* (Table S13, Figure S13).................16

Treatment effect on territory defence in *A. percula* (Table S14, Figure S14).......................................17

Treatment effect on territory defence in *A. perideraion* (Table S15, Figure S15)…………….……...18

Treatment effect on territory defence in *A. clarkii* (Table S16, Figure S16).........................................19

Treatment effect on anemone maintenance in *A. percula* (Table S17, Figure S17)..............................20

Treatment effect on anemone maintenance in *A. perideraion* (Table S18, Figure S18)……….…..…21

Treatment effect on anemone maintenance in *A. clarkii* (Table S19, Figure S19)................................22

| **Glossary of terms and summary statistics for model summary tables** | |
| --- | --- |
| **Term** | **Description** |
| ***percula*** | *Amphiprion* percula (anemonefish species) |
| ***perideraion*** | *Amphiprion* *perideraion* (anemonefish species) |
| ***clarkii*** | *Amphiprion* *clarkii* (anemonefish species) |
| ***R1*** | Rank 1 (dominant female) |
| ***R2*** | Rank 2 (dominant male) |
| ***BAS*** | Baseline condition (no manipulation) |
| ***CON*** | Control condition (focal fish caught and released, empty box present) |
| ***MAN*** | Manipulation condition (helping prevention by confinement to box) |
| **size.ratio_z** | z-standardised (mean = 0, SD = 1) size ratio between focal subordinate and immediate dominant (i.e., rank 2) |
| ***SR_closer*** | Closer in size (z > 0) to immediate dominant (size.ratio_z level) |
| ***SR_further*** | Further in size (z < 0) to immediate dominant (size.ratio_z level) |
| **Δ** | Difference in response (proportion of time displaying behaviour across observation) between contrast groups |
| **Median Δ** | Posterior median of the difference in response. |
| **89% HDI** | 89% highest density interval (i.e., range of most credible values of parameter) |
| **Median %** | Median percentage difference between groups, calculated relative to reference group (right side of contrast). |
| **Pr(Δ > 0)** | Posterior probability that the difference in response is greater than zero |

**Table S1**:

Anemonefish behaviour ethogram

| **Category** | **Type** | **Behaviour** | **Description** |
| --- | --- | --- | --- |
| **Aggression** | Overt | Chase | Rapidly swims directly towards conspecific |
|  |  | Bite | Bites conspecific |
|  |  | Jerk | Short and rapid head-jerk towards conspecific when in close proximity |
|  |  | Flick | Rapidly darts and turns, flicking their caudal fin at conspecific |
|  | Display | Aggressive display | Fish meets another, turns side on with erect fins and stiffened body posture |
| **Proactive submission**  *(not direct response to aggression)* | Avoidance | Moving away | Rapidly swims out of the way of a passing conspecific |
|  | Display | Body shake | Shakes body side to side in jolty fashion |
|  |  | S bend | Displays S-shaped posture |
|  |  | C bend | Displays C-shaped posture |
| **Helping** | Anemone maintenance | Bite | Biting the mouth, tentacles or foot of anemone |
|  |  | Massage | Moving vigorously against anemone foot |
|  |  | Clean | Picking up sand or debris and spits it out outside anemone. |
|  |  | Aerate | Swimming vigorously through anemone tentacles |
|  | Defence | Chase | Rapidly swims towards predator/competitor |
|  |  | Bite | Bites predator/competitor |
|  |  | Guard | Gaze following predator/competitor, often bobbing up-down or side-to-side |
|  |  | Head down display | Face head downwards, often swimming upwards in this position |
|  |  | Flick | Rapidly darts and turns, flicking their caudal fin at predator or competitor |
|  |  | Jerk | Short and rapid head-jerk towards conspecific when in close proximity |

Adapted from Rueger et al. (2022). Aggression and proactive submission behaviours are directed towards conspecifics and defence behaviours are directed towards heterospecifics, either food competitors (such as other Pomacentridae) or predators (egg predators such as Labridae, anemone predators such as Chaetodontidae, or larger fish that may predate anemonefish such as Labridae, Epinephelinae and Lutjanidae).

**
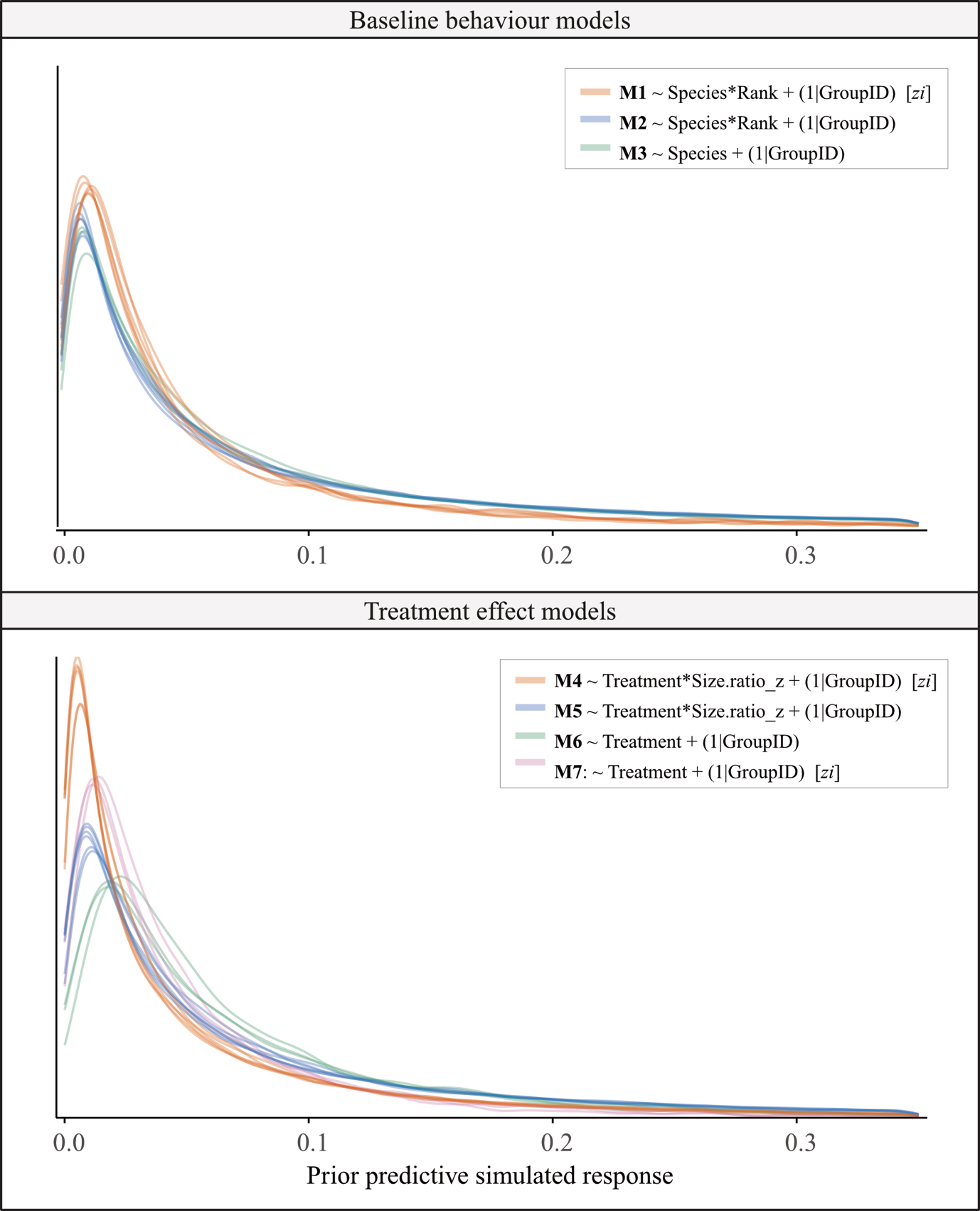
**

**Figure S1. Prior predictive distributions for all baseline and treatment-effect model structures.** Lines show simulated responses from all final model structures. Multiple lines within each model represent predictions across predictor levels (species, species × rank, treatment, or treatment × size.ratio_z); for treatment × size.ratio_z, lines are shown for each treatment, for SR_closer and SR_further. Priors: conditional intercept ~ Normal(−3, 1); zero-inflation intercept ~ Normal(−1, 1); fixed effects ~ Normal(0, 1); interactions ~ Normal(0, 0.5); dispersion (ϕ) ~ Gamma(4, 0.1). Model–response mapping: **M1** = baseline aggression; **M2** = baseline submission; **M3** = baseline helping; **M4** = *percula* (aggression, display, defence) and *perideraion* (defence, maintenance); **M5** = *percula* (avoidance, display) and *perideraion* (aggression, display, avoidance); **M6** = *clarkii* (aggression, avoidance); **M7** = *clarkii* (display, defence, maintenance).

**Table S2**:

Species comparison of baseline aggression from dominants (rank 1 and 2) towards focal subordinates

| **Contrast** | **Median Δ** | **89% HDI (Δ)** | **Median %** | | **89% HDI (%)** | **Pr(Δ>0)** |
| --- | --- | --- | --- | --- | --- | --- |
| **Species comparisons:** |  |  |  | |  |  |
| *percula—clarkii* | -0.0057 | [-0.0111, -0.0006] | | -55.17 | [-82.11, -21.80] | 0.030 |
| *perideraion—clarkii* | 0.0102 | [0.0027, 0.0178] | | 99.77 | [3.37, 224.02] | 0.983 |
| *perideraion—percula* | 0.0159 | [0.0097, 0.022] | | 349.01 | [106.33, 632.62] | >.999 |

| **Rank comparisons within species:** | |  |  |  |  |
| --- | --- | --- | --- | --- | --- |
| *clarkii: R2 – R1* | 0.0043 | [-0.0023, 0.0118] | 55.93 | [-81.96, -23.64] | 0.863 |
| *percula: R2 – R1* | 0.0016 | [-0.0014, 0.0048] | 46.31 | [0.48, 214.83] | 0.827 |
| *perideraion: R2 – R1* | 0.0110 | [0.0017, 0.0203] | 73.64 | [116.27, 625.79] | 0.973 |

Bayesian pairwise contrasts derived from a zero-inflated beta mixed-effects model: Aggression (proportion of time visible) ~ species*rank + (1|groupID). Contrasts represent posterior differences between species (marginalised across rank) and between ranks within species.

**
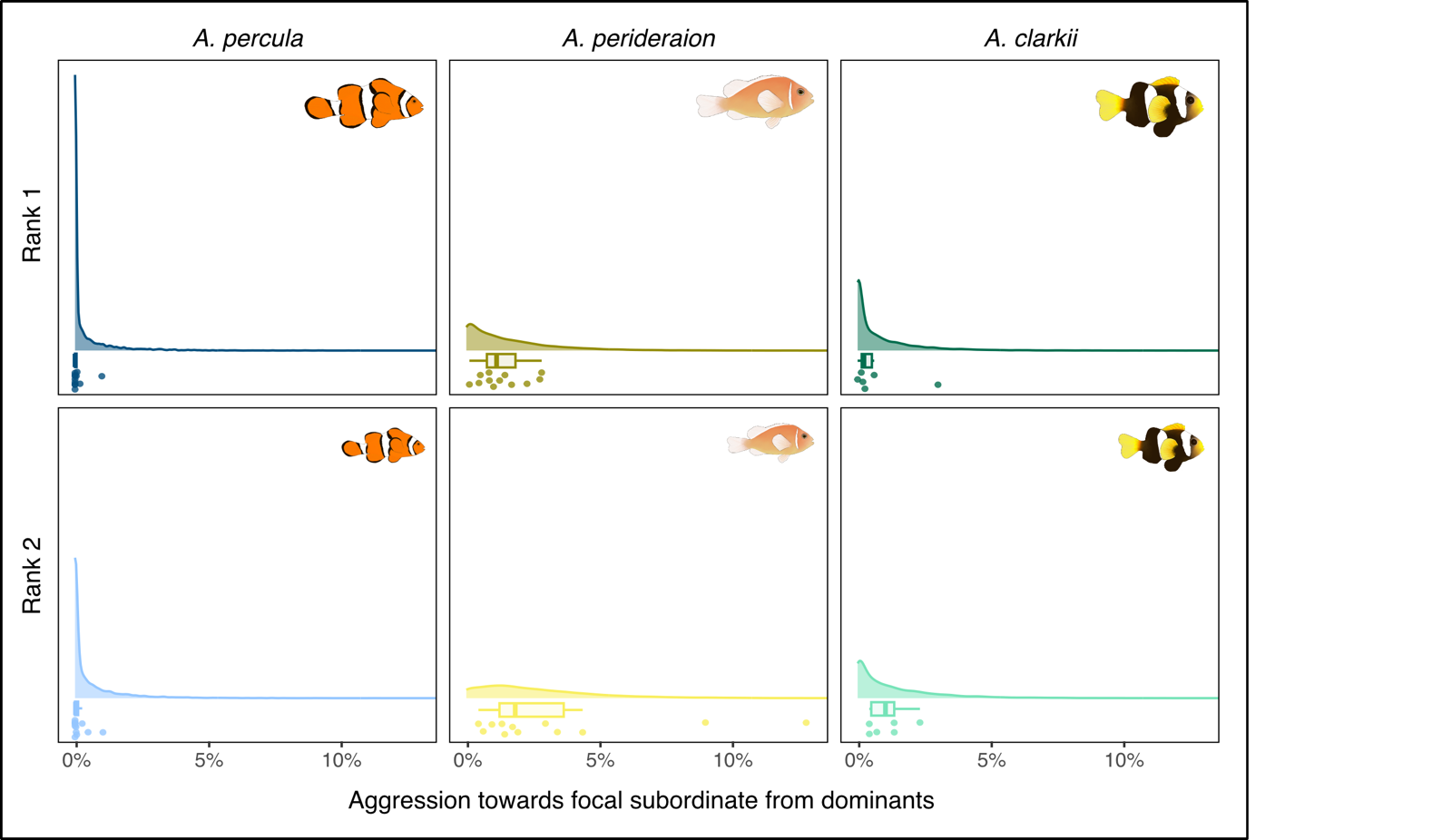
**

**Figure S2. Baseline aggression from dominants toward focal subordinates by species and rank.** Curves show posterior predicted distributions of aggression (percentage of time visible) from the fitted model. Boxplots summarize observed values and points represent individual observations.

**Table S3**:

Species comparison of baseline proactive submission (displays and avoidance) towards dominants (rank 1 and 2) by focal subordinates

| **Contrast** | **Median Δ** | **89% HDI (Δ)** | | **Median %** | **89% HDI (%)** | **Pr(Δ>0)** |
| --- | --- | --- | --- | --- | --- | --- |
| **Species comparisons:** |  |  | |  |  |  |
| *percula—clarkii* | 0.0083 | | [0.0043, 0.0125] | 112.85 | [33.02, 204.84] | 0.999 |
| *perideraion—clarkii* | 0.0109 | | [0.0063, 0.0156] | 147.20 | [57.07, 257.15] | >.999 |
| *perideraion—percula* | 0.0026 | | [-0.0028, 0.0074] | 16.19 | [-15.85, 51.90] | 0.796 |

| **Rank comparisons within species:** | |  | | |  |  | |  |
| --- | --- | --- | --- | --- | --- | --- | --- | --- |
| *clarkii: R2 – R1* | 0.0020 | | [-0.0024, 0.006] | 32.46 | | [-32.94, 108.65] | 0.797 | |
| *percula: R2 – R1* | -0.0005 | | [-0.0056, 0.0049] | -3.17 | | [-31.99, 31.60] | 0.439 | |
| *perideraion: R2 – R1* | -0.0018 | | [-0.0083, 0.0039] | -9.51 | | [-37.93, 21.44] | 0.317 | |

| **Effect of size ratio** | |  |  |  |  |
| --- | --- | --- | --- | --- | --- |
| size.ratio_*z* | 0.0556 | [-0.0910, 0.1960] | - | - | 0.738 |

Bayesian pairwise contrasts derived from a beta mixed-effects model: Proactive submission (proportion of time visible) ~ species*rank + size.ratio_z + (1|groupID). Contrasts represent posterior differences between species and between ranks within species.

**
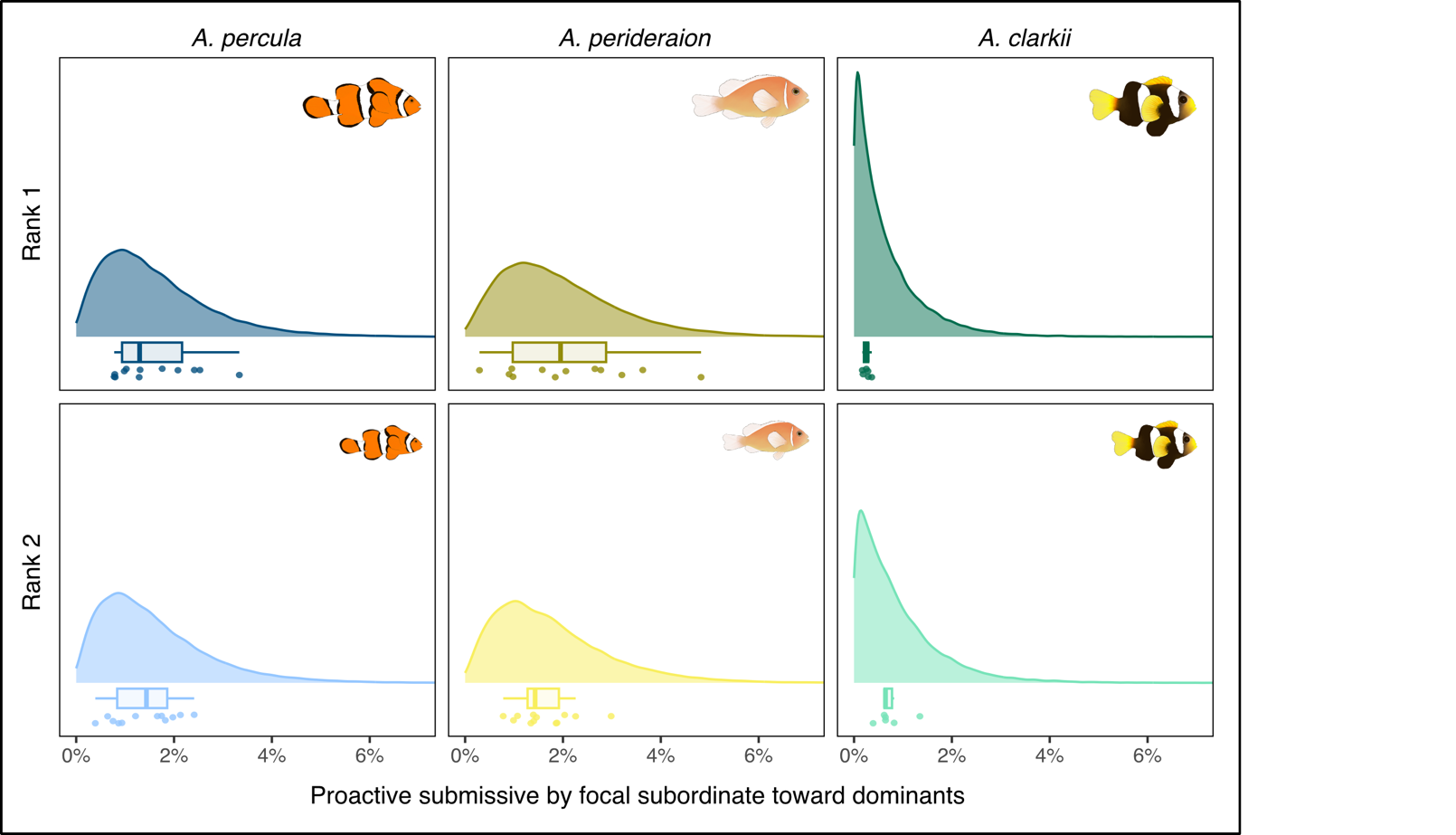
**

**Figure S3. Baseline proactive submission from focal subordinates toward dominants by species and rank.** Curves show posterior predicted distributions of aggression (percentage of time visible) from the fitted model. Boxplots summarize observed values and points represent individual observations.

**Table S4**:

Species comparison of baseline helping behavior (anemone maintenance, territory defence) by focal subordinates

| **Contrast/Term** | **Median Δ** | **89% HDI (Δ)** | **Median %** | **89% HDI (%)** | **Pr(Δ>0)** |
| --- | --- | --- | --- | --- | --- |
| **Species comparisons:** |  |  |  |  |  |
| *percula—clarkii* | 0.0454 | [0.0342, 0.0589] | 391.03 | [169.91, 675.97] | >.999 |
| *perideraion—clarkii* | 0.0033 | [-0.0027, 0.0108] | 28.93 | [-31.76, 110.15] | 0.764 |
| *perideraion—percula* | -0.0423 | [-0.0522, -0.0298] | -73.86 | [-83.07, -62.28] | <.001 |

| **Effect of size ratio** | |  |  |  |  |
| --- | --- | --- | --- | --- | --- |
| size.ratio_*z* | 0.2078 | [0.0208, 0.3764] | - | - | 0.934 |

Bayesian pairwise contrasts derived from a beta mixed-effects model: helping behavior (as a proportion of time visible) ~ species + size.ratio_z. Contrasts represent posterior differences between species and between ranks within species.


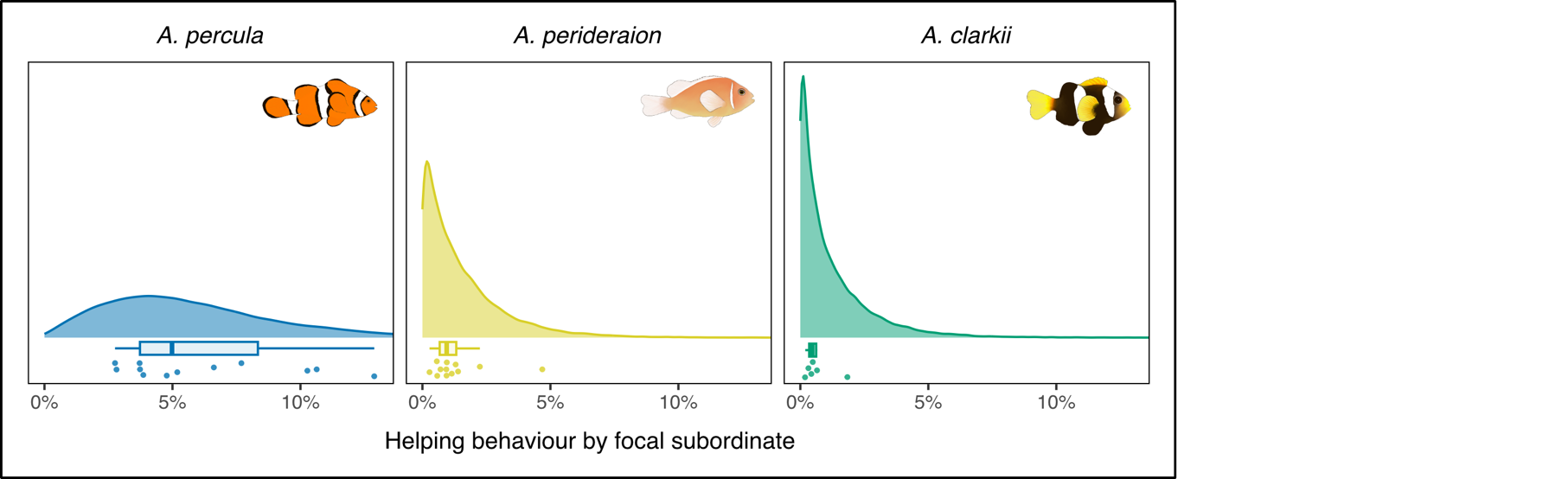


**Figure S4. Baseline cooperative behaviour (anemone maintenance and defence) of focal subordinate by species.** Curves show posterior predicted distributions of aggression (percentage of time visible) from the fitted model. Boxplots summarize observed values and points represent individual observations.

**Table S5**:

Treatment effects on aggression towards the focal subordinate in *Amphiprion percula*.

| **Contrast/Term** | **Median Δ** | **89% HDI (Δ)** | **Median %** | **89% HDI (%)** | **Pr(Δ>0)** |
| --- | --- | --- | --- | --- | --- |
| **Treatment comparisons:** |  |  |  |  |  |
| *CON—BAS* | -0.0011 | [-0.005, 0.0027] | -24.89 | [-81.52, 46.38] | 0.292 |
| *MAN—BAS* | 0.0048 | [-0.001, 0.0106] | 100.68 | [-21.77, 256.24] | 0.939 |
| *MAN—CON* | 0.006 | [-4e-04, 0.0127] | 170.09 | [-42.91, 499.37] | 0.949 |

| **Treatment comparisons within each size ratio level:** | | |  |  |  |
| --- | --- | --- | --- | --- | --- |
| *SR_ closer: CON—BAS* | -0.0019 | [-0.0066, 0.0024] | -35.39 | [-84.57, 28.09] | 0.212 |
| *SR_closer: MAN—BAS* | 0.0078 | [-0.0004, 0.0168] | 134.20 | [-15.11, 338.14] | 0.957 |
| *SR_closer: MAN—CON* | 0.0098 | [0.001, 0.0196] | 268.91 | [-30.43, 741.73] | 0.981 |
| *SR_further: CON—BAS* | -0.0005 | [-0.0048, 0.0039] | -17.62 | [-87.85, 95.20] | 0.403 |
| *SR_further: MAN—BAS* | 0.0007 | [-0.0031, 0.0055] | 23.56 | [-65.4, 166.31] | 0.652 |
| *SR_further: MAN—CON* | 0.0013 | [-0.004, 0.0068] | 51.58 | [-83.68, 330.19] | 0.693 |

| **Effect of size ratio:** | | |  |  |  |
| --- | --- | --- | --- | --- | --- |
| size.ratio_*z* | 0.126682975 | [-0.2882, 0.5135] | - | - | 0.6918 |

Bayesian pairwise contrasts derived from a zero-inflated beta mixed-effects model: Aggression (proportion of time visible) ~ treatment*size.ratio_z + (1|groupID). Contrasts represent posterior differences between treatments (marginalised over size.ratio_z) and between treatments within each size.ratio_z level.


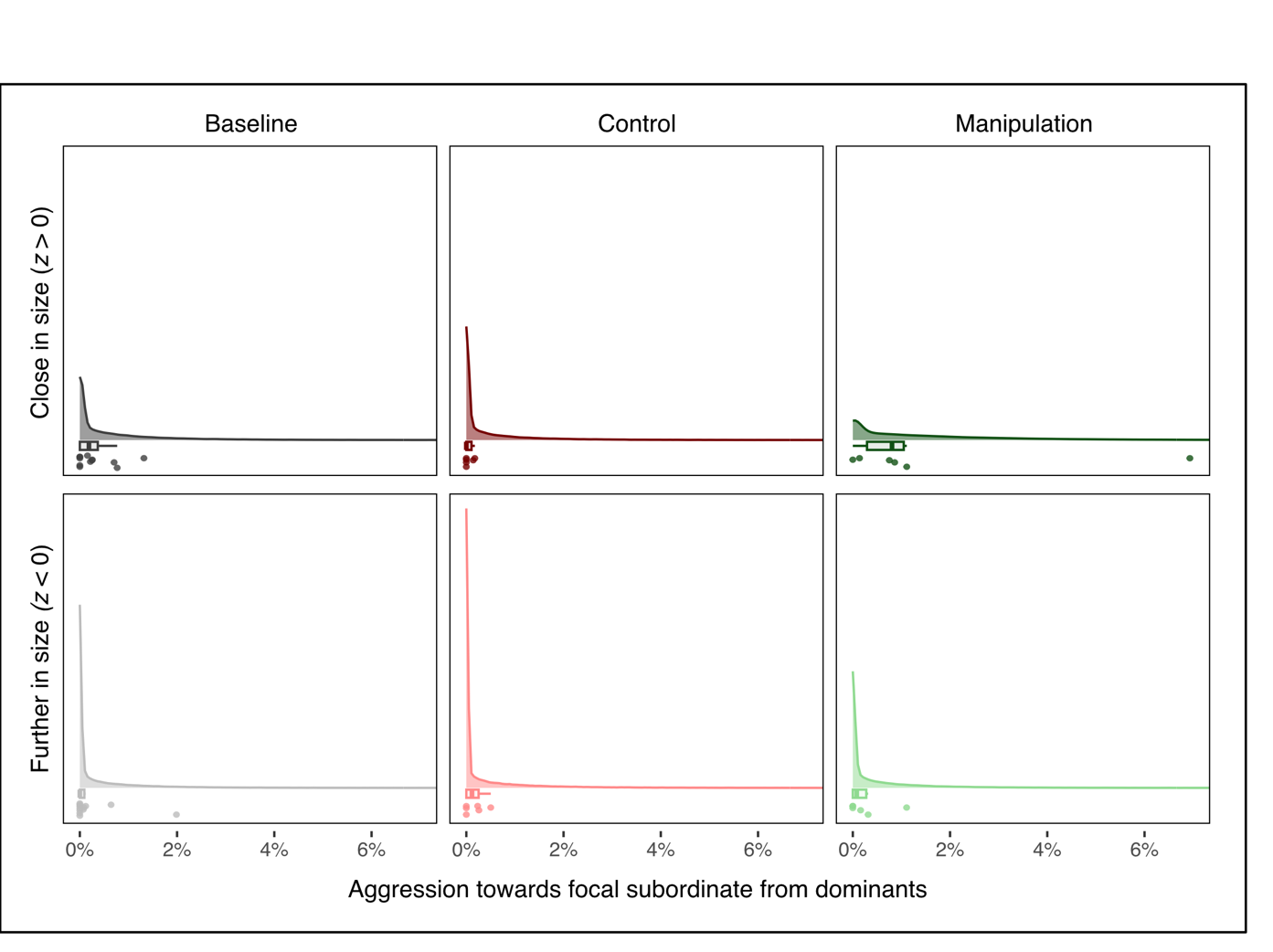


**Figure S5. Aggression towards the focal subordinate across conditions (baseline, control, treatment) at each size ratio level (close in size, further in size) in *Amphiprion percula*.** Curves show posterior predicted distributions of aggression (percentage of time visible) from the fitted model. Boxplots summarize observed values and points represent individual observations.

**Table S6**:

Treatment effects on aggression towards the focal subordinate in *Amphiprion perideraion*.

| **Contrast/Term** | **Median Δ** | **89% HDI (Δ)** | **Median %** | **89% HDI (%)** | **Pr(Δ>0)** |
| --- | --- | --- | --- | --- | --- |
| **Treatment comparisons:** |  |  |  |  |  |
| *CON—BAS* | -0.0257 | [-0.0421, -0.0106] | -56.04 | [-77.02, -30.65] | 0.004 |
| *MAN—BAS* | 0.0361 | [0.0083, 0.065] | 77.86 | [12.61, 145.51] | 0.989 |
| *MAN—CON* | 0.0616 | [0.0326, 0.092] | 303.54 | [91.69, 564.85] | >.999 |

| **Treatment comparisons within each size ratio level:** | | |  |  |  |
| --- | --- | --- | --- | --- | --- |
| *SR_closer: CON—BAS* | -0.0263 | [-0.0415, -0.0111] | -58.59 | [-79.43, -35.89] | 0.002 |
| *SR_closer: MAN—BAS* | 0.0144 | [-0.0074, 0.0368] | 31.86 | [-19.24, 84.33] | 0.868 |
| *SR_closer: MAN—CON* | 0.0408 | [0.0188, 0.0632] | 217.72 | [59.74, 426.38] | 0.999 |
| *SR_further: CON—BAS* | -0.0255 | [-0.0464, -0.0057] | -55.82 | [-81.89, -22.01] | 0.020 |
| *SR_further: MAN—BAS* | 0.0459 | [0.0107, 0.0845] | 98.82 | [13.18, 194.83] | 0.987 |
| *SR_further: MAN—CON* | 0.0713 | [0.0331, 0.112] | 352.91 | [58.03, 734.97] | >.999 |

| **Effect of size ratio:** | | |  |  |  |
| --- | --- | --- | --- | --- | --- |
| size.ratio_*z* | -0.0130 | [-0.2887, 0.2538] | - | - | 0.4685 |

Bayesian pairwise contrasts derived from a beta mixed-effects model: Aggression (proportion of time visible) ~ treatment*size.ratio_z + (1|groupID). Contrasts represent posterior differences between treatments (marginalised over size.ratio_z) and between treatments within each size.ratio_z level.


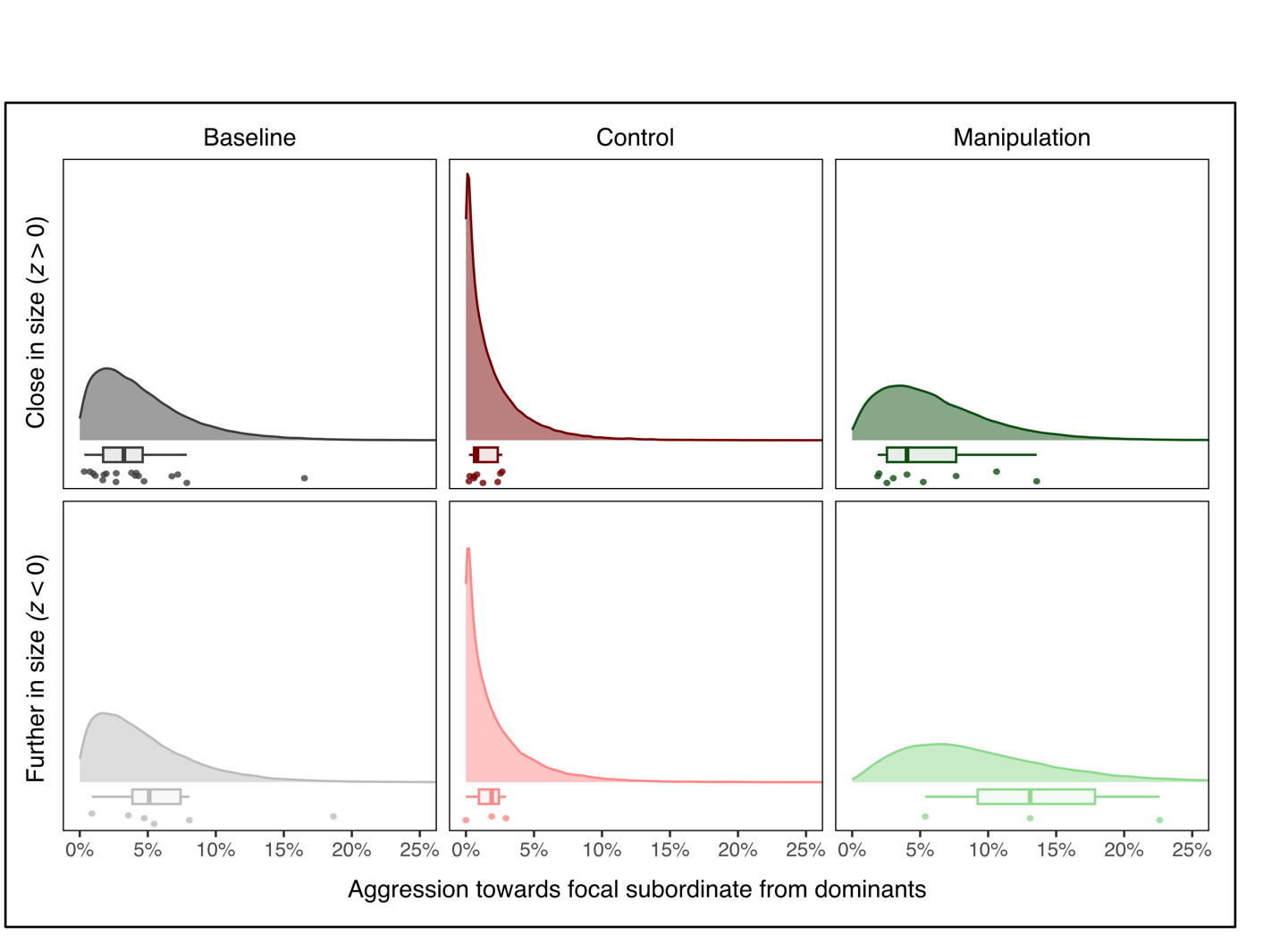


**Figure S6. Aggression towards the focal subordinate across conditions (baseline, control, treatment) at each size ratio level (closer in size, further in size) in *Amphiprion perideraion*.** Curves show posterior predicted distributions of aggression (percentage of time visible) from the fitted model. Boxplots summarize observed values and points represent individual observations.

**Table S7**:

Treatment effects on aggression towards the focal subordinate in *Amphiprion clarkii*.

| **Contrast/Term** | **Median Δ** | **89% HDI (Δ)** | **Median %** | **89% HDI (%)** | **Pr(Δ>0)** |
| --- | --- | --- | --- | --- | --- |
| **Treatment comparisons:** |  |  |  |  |  |
| *CON—BAS* | -0.0072 | [-0.0167, 0.0022] | -36.2341 | [-72.78, 1.87] | 0.102 |
| *MAN—BAS* | 0.0040 | [-0.0083, 0.0166] | 19.8554 | [-41.85, 84.45] | 0.713 |
| *MAN—CON* | 0.0111 | [-0.0018, 0.0246] | 87.9286 | [-22.38, 232.70] | 0.932 |

Bayesian pairwise contrasts derived from a beta mixed-effects model: Aggression (proportion of time visible) ~ treatment + (1|groupID). Contrasts represent posterior differences between treatments.


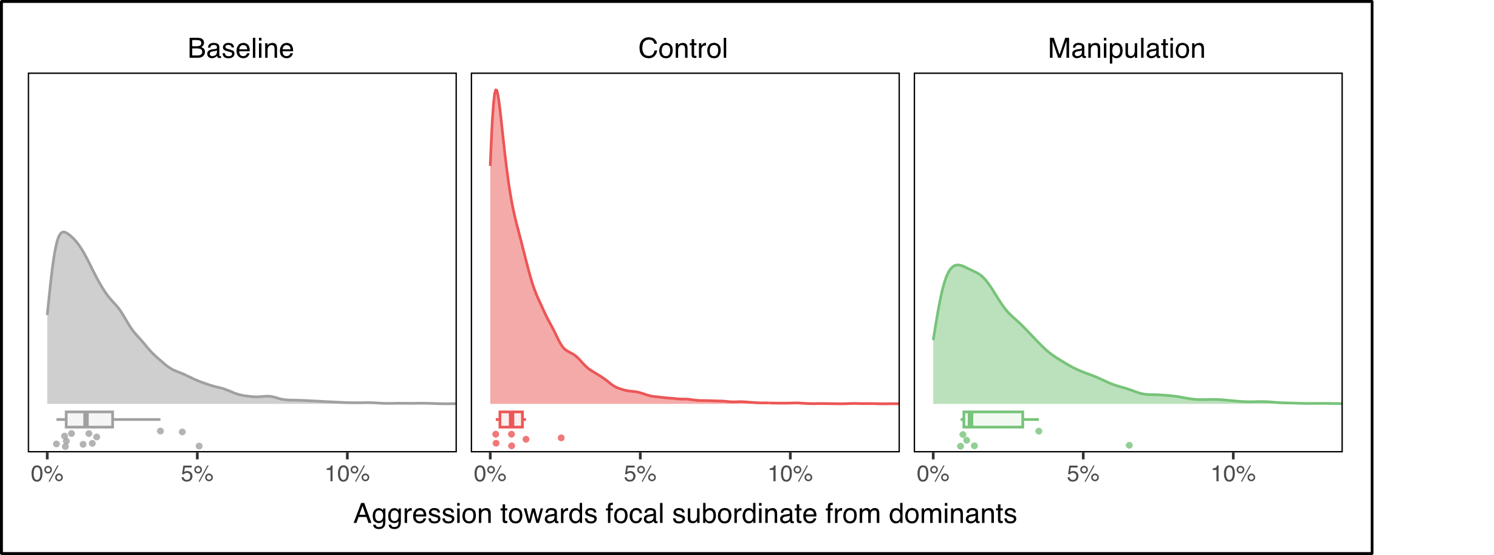


**Figure S7. Aggression towards the focal subordinate across conditions in *Amphiprion clarkii*.** Curves show posterior predicted distributions of aggression (percentage of time visible) from the fitted model. Boxplots summarize observed values and points represent individual observations.

**Table S8**:

Treatment effects on proactive submissive displays by focal subordinate in *Amphiprion percula*.

| **Contrast/Term** | **Median Δ** | **89% HDI (Δ)** | **Median %** | **89% HDI (%)** | **Pr(Δ>0)** |
| --- | --- | --- | --- | --- | --- |
| **Treatment comparisons:** |  |  |  |  |  |
| *CON—BAS* | -0.0001 | [-0.0048, 0.0048] | -0.73 | [-53.32, 61.39] | 0.492 |
| *MAN—BAS* | 0.0062 | [0.0007, 0.0118] | 78.33 | [-3.00, 163.77] | 0.971 |
| *MAN—CON* | 0.0062 | [-0.0002, 0.0131] | 78.68 | [-22.61, 202.09] | 0.936 |

| **Treatment comparisons within each size ratio level:** | | |  |  |  |
| --- | --- | --- | --- | --- | --- |
| *SR_closer: CON—BAS* | -0.0015 | [-0.007, 0.0046] | -16.91 | [-68.53, 43.15] | 0.329 |
| *SR_closer: MAN—BAS* | 0.0088 | [0.0005, 0.0172] | 94.84 | [-6.40, 203.39] | 0.969 |
| *SR_closer: MAN—CON* | 0.0103 | [0.0011, 0.0195] | 134.43 | [-17.66, 336.29] | 0.970 |
| *SR_further: CON—BAS* | 0.0011 | [-0.0043, 0.0072] | 17.00 | [-64.87, 112.45] | 0.628 |
| *SR_further: MAN—BAS* | 0.003 | [-0.0027, 0.0092] | 48.00 | [-41.58, 153.21] | 0.821 |
| *SR_further: MAN—CON* | 0.0019 | [-0.006, 0.0095] | 26.04 | [-66.76, 159.29] | 0.663 |

| **Effect of size ratio:** | | |  |  |  |
| --- | --- | --- | --- | --- | --- |
| size.ratio_*z* | 0.124483185 | [-0.149, 0.3985] | - | - | 0.7735 |

Bayesian pairwise contrasts derived from a zero-inflated beta mixed-effects model: Proactive submissive displays (proportion of time visible) ~ treatment*size.ratio_z + (1|groupID). Contrasts represent posterior differences between treatments (marginalised over size.ratio_z) and between treatments within each size.ratio_z level.


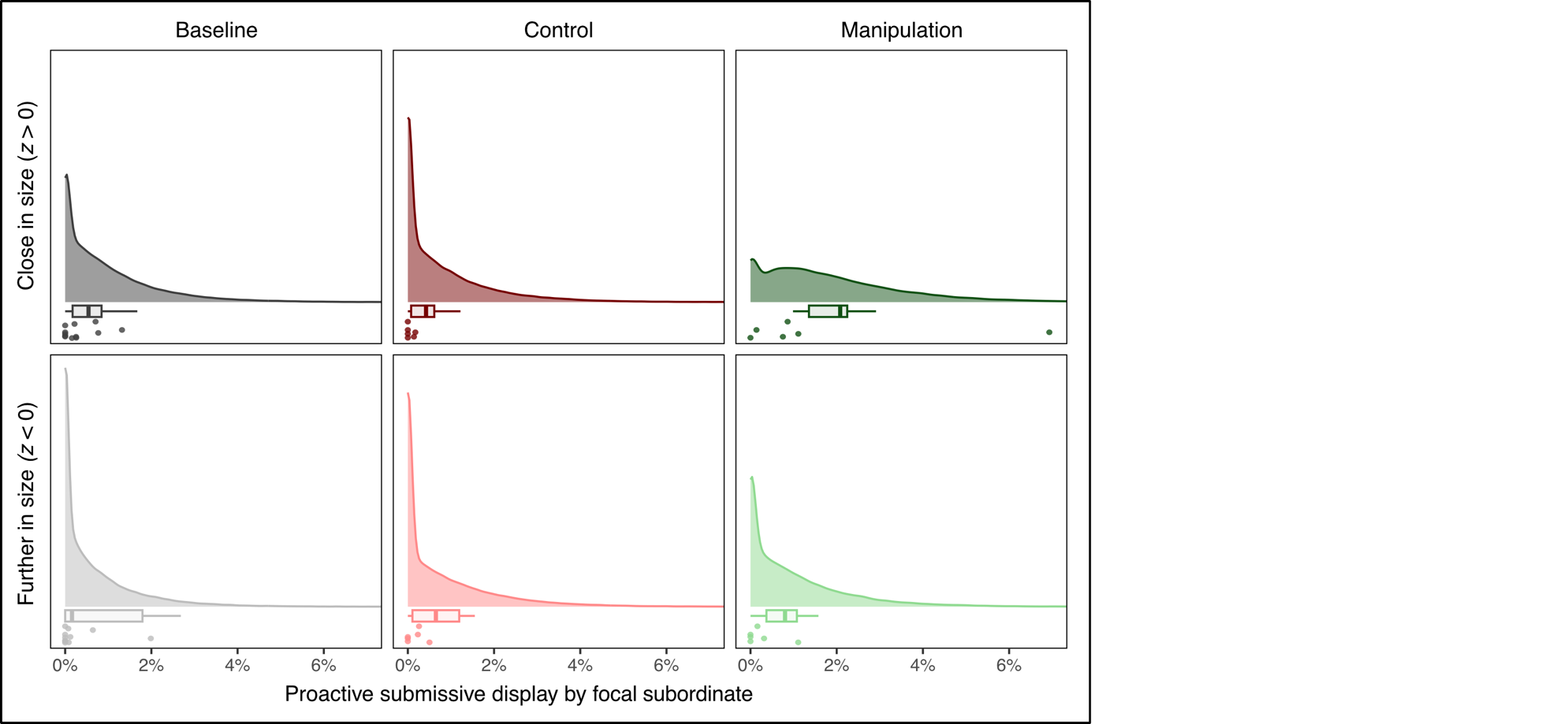


**Figure S8. Proactive submissive displays towards the focal subordinate across conditions (baseline, control, treatment) at each size ratio level (close in size, further in size) in *Amphiprion percula*.** Curves show posterior predicted distributions of displays (percentage of time visible) from the fitted model. Boxplots summarize observed values and points represent individual observations.

**Table S9**:

Treatment effects on proactive submissive displays by focal subordinate in *Amphiprion perideraion*.

| **Contrast/Term** | **Median Δ** | **89% HDI (Δ)** | **Median %** | **89% HDI (%)** | **Pr(Δ>0)** |
| --- | --- | --- | --- | --- | --- |
| **Treatment comparisons:** |  |  |  |  |  |
| *CON—BAS* | -0.0007 | [-0.0077, 0.0066] | -4.84 | [-49.01, 47.57] | 0.438 |
| *MAN—BAS* | 0.0075 | [5e-04, 0.0145] | 53.39 | [-0.36, 111.97] | 0.964 |
| *MAN—CON* | 0.0081 | [-0.001, 0.0171] | 61.44 | [-21.02, 159.79] | 0.916 |

| **Treatment comparisons within each size ratio level:** | | |  |  |  |
| --- | --- | --- | --- | --- | --- |
| *SR_closer: CON—BAS* | -0.0061 | [-0.0116, -5e-04] | -39.61 | [-67.31, -8.98] | 0.046 |
| *SR_closer: MAN—BAS* | 0.0137 | [0.0044, 0.0237] | 88.47 | [18.41, 165.57] | 0.992 |
| *SR_closer: MAN—CON* | 0.0198 | [0.0096, 0.0297] | 211.85 | [61.89, 394.03] | 0.999 |
| *SR_further: CON—BAS* | 0.0018 | [-0.0075, 0.0117] | 13.60 | [-58.54, 88.72] | 0.616 |
| *SR_further: MAN—BAS* | 0.0031 | [-0.0047, 0.0115] | 23.58 | [-37.88, 91.73] | 0.741 |
| *SR_further: MAN—CON* | 0.0013 | [-0.0104, 0.0125] | 8.99 | [-62.61, 103.29] | 0.663 |

| **Effect of size ratio:** | | |  |  |  |
| --- | --- | --- | --- | --- | --- |
| size.ratio_*z* | 0.093363319 | [-0.1378, 0.3258] | - | - | 0.747 |

Bayesian pairwise contrasts derived from a zero-inflated beta mixed-effects model: Proactive submissive displays (proportion of time visible) ~ treatment*size.ratio_z + (1|groupID). Contrasts represent posterior differences between treatments (marginalised over size.ratio_z) and between treatments within each size.ratio_z level.


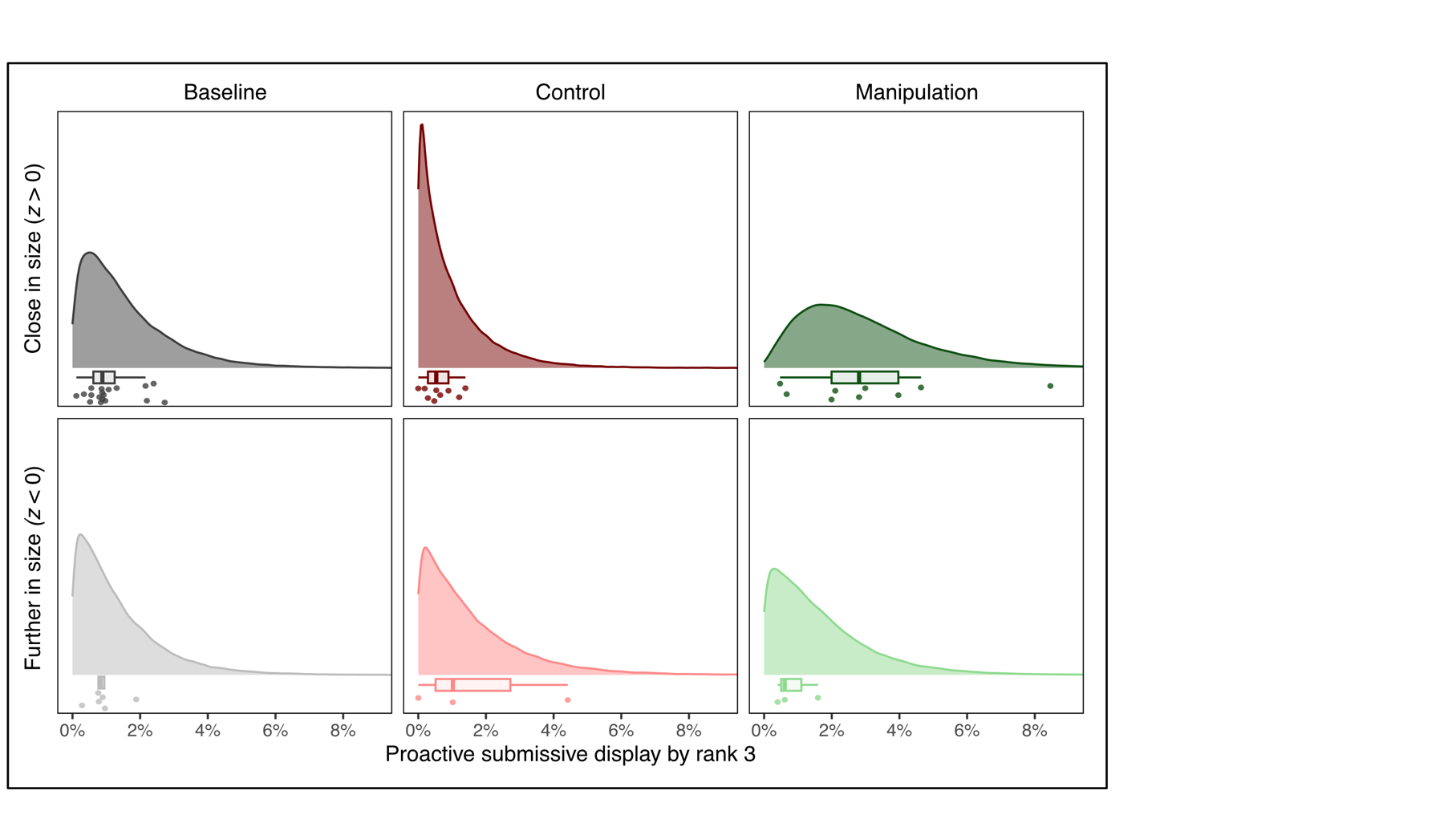


**Figure S9. Proactive submissive displays towards the focal subordinate across conditions (baseline, control, treatment) at each size ratio level (close in size, further in size) in *Amphiprion perideraion*.** Curves show posterior predicted distributions of displays (percentage of time visible) from the fitted model. Boxplots summarize observed values and points represent individual observations.

**Table S10**:

Treatment effects on proactive submissive displays by focal subordinate in *Amphiprion clarkii*.

| **Contrast/Term** | **Median Δ** | **89% HDI (Δ)** | **Median %** | **89% HDI (%)** | **Pr(Δ>0)** |
| --- | --- | --- | --- | --- | --- |
| **Treatment comparisons:** |  |  |  |  |  |
| *CON—BAS* | -0.001 | [-0.007, 0.0055] | -14.04 | [-77.56, 66.82] | 0.391 |
| *MAN—BAS* | 0.0005 | [-0.0056, 0.0073] | 7.17 | [-66.07, 100.36] | 0.557 |
| *MAN—CON* | 0.0015 | [-0.0066, 0.0099] | 24.84 | [-75.87, 204.91] | 0.632 |

Bayesian pairwise contrasts derived from a beta mixed-effects model: Proactive submissive displays (proportion of time visible) ~ treatment + (1|groupID). Contrasts represent posterior differences between treatments.


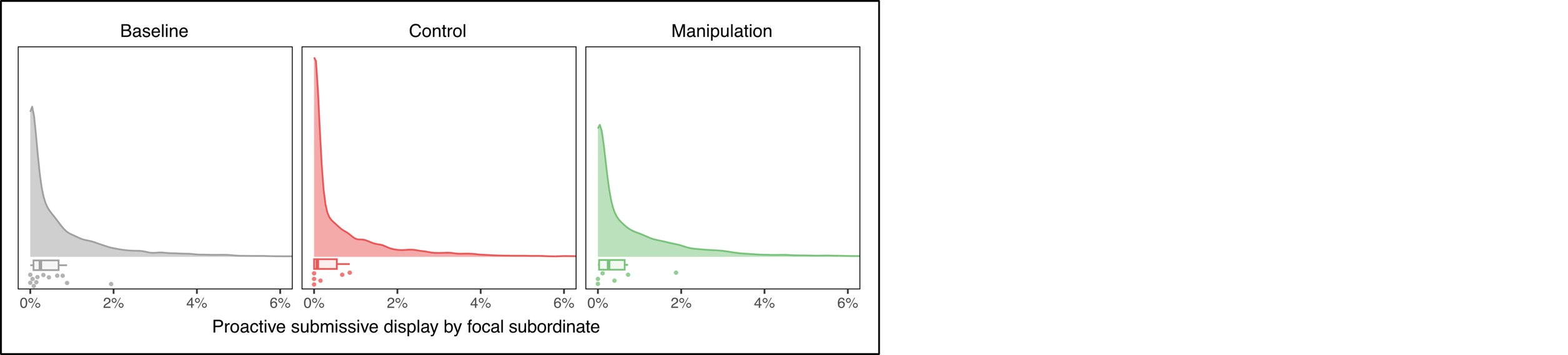


**Figure S10. Proactive submissive displays towards the focal subordinate across conditions (baseline, control, treatment) in *Amphiprion clarkii*.** Curves show posterior predicted distributions of displays (percentage of time visible) from the fitted model. Boxplots summarize observed values and points represent individual observations.

**Table S11**:

Treatment effects on proactive submissive avoidance by focal subordinate in *Amphiprion percula*.

| **Contrast/Term** | **Median Δ** | **89% HDI (Δ)** | **Median %** | **89% HDI (%)** | **Pr(Δ>0)** |
| --- | --- | --- | --- | --- | --- |
| **Treatment comparisons:** |  |  |  |  |  |
| *CON—BAS* | -0.0038 | [-0.0113, 0.0029] | -16.24 | [-43.62, 11.64] | 0.191 |
| *MAN—BAS* | 0.0024 | [-0.0051, 0.01] | 10.20 | [-21.36, 43.53] | 0.703 |
| *MAN—CON* | 0.0061 | [-0.0022, 0.0148] | 31.39 | [-13.81, 84.57] | 0.885 |

| **Treatment comparisons within each size ratio level:** | | |  |  |  |
| --- | --- | --- | --- | --- | --- |
| *SR_closer: CON—BAS* | -0.0065 | [-0.0148, 0.0022] | -26.70 | [-56.42, 4.36] | 0.111 |
| *SR_closer: MAN—BAS* | 0.0041 | [-0.0061, 0.0142] | 16.77 | [-25.12, 60.17] | 0.742 |
| *SR_closer: MAN—CON* | 0.0105 | [-9e-04, 0.0213] | 58.74 | [-12.09, 138.26] | 0.942 |
| *SR_further: CON—BAS* | -0.0018 | [-0.0117, 0.0076] | -8.13 | [-47.48, 33.99] | 0.379 |
| *SR_further: MAN—BAS* | 0.0005 | [-0.0093, 0.0098] | 2.11 | [-38.52, 44.98] | 0.533 |
| *SR_further: MAN—CON* | 0.0023 | [-0.0085, 0.0139] | 11.62 | [-40.94, 72.71] | 0.633 |

| **Effect of size ratio:** | | |  |  |  |
| --- | --- | --- | --- | --- | --- |
| size.ratio_*z* | 0.0495 | [-0.1699, 0.2756] | - | - | 0.653 |

Bayesian pairwise contrasts derived from beta mixed-effects model: Proactive submissive avoidance (proportion of time visible) ~ treatment*size.ratio_z + (1|groupID). Contrasts represent posterior differences between treatments (marginalised over size.ratio_z) and between treatments within each size.ratio_z level.

*
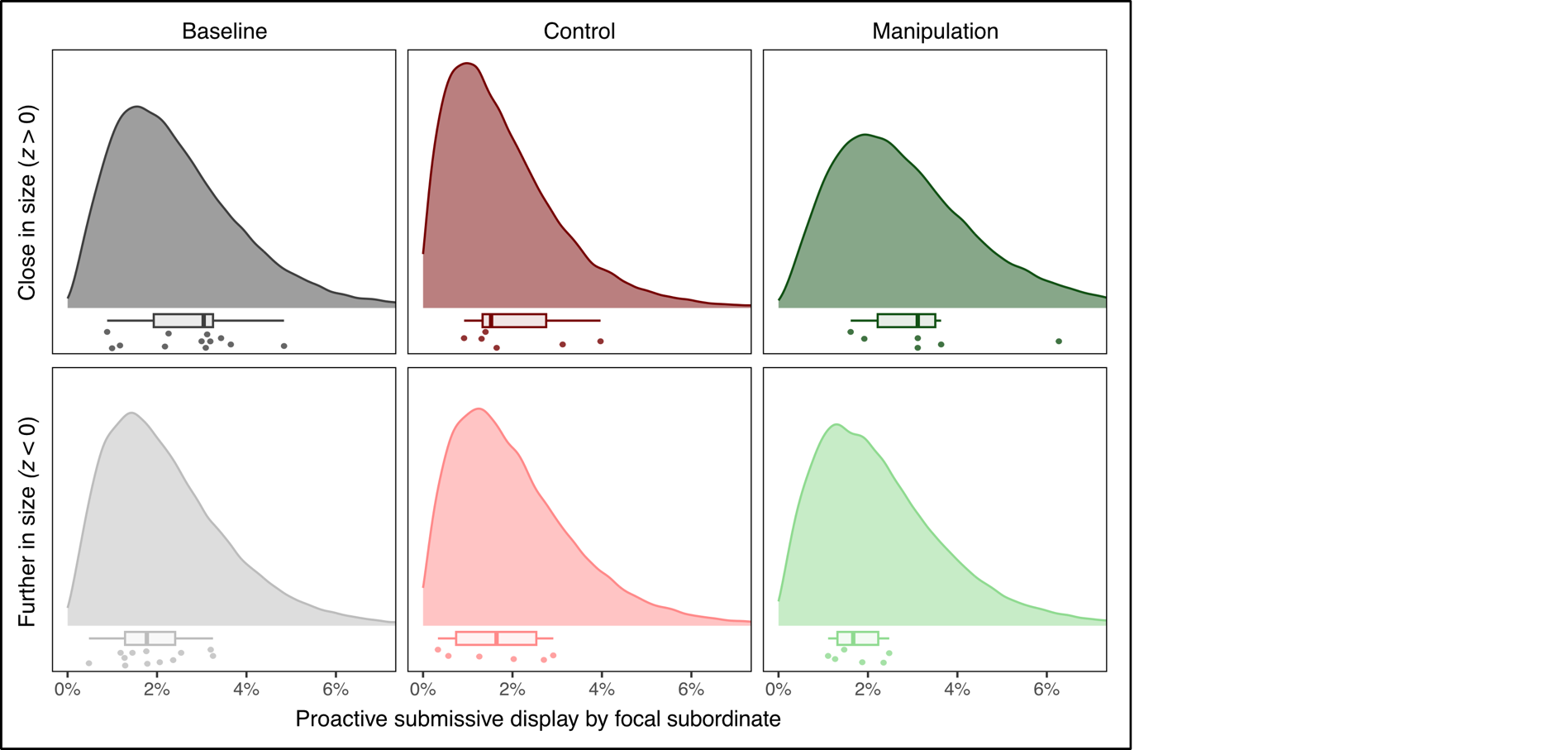
*

**Figure S11. Proactive submissive avoidance towards the focal subordinate across conditions (baseline, control, treatment) at each size ratio level (close in size, further in size) in *Amphiprion percula*.** Curves show posterior predicted distributions of avoidance (percentage of time visible) from the fitted model. Boxplots summarize observed values and points represent individual observations.

**Table S12**:

Treatment effects on proactive submissive avoidance by focal subordinate in *Amphiprion percula*.

| **Contrast/Term** | **Median Δ** | **89% HDI (Δ)** | **Median %** | **89% HDI (%)** | **Pr(Δ>0)** |
| --- | --- | --- | --- | --- | --- |
| **Treatment comparisons:** |  |  |  |  |  |
| *CON—BAS* | 0.0003 | [-0.012, 0.0138] | 1.0787 | [-38.4, 51.7466] | 0.517 |
| *MAN—BAS* | 0.0027 | [-0.0087, 0.0136] | 9.6054 | [-31.43, 47.7346] | 0.649 |
| *MAN—CON* | 0.0022 | [-0.0129, 0.0174] | 7.8486 | [-43.48, 63.7033] | 0.588 |

| **Treatment comparisons within each size ratio level:** | | |  |  |  |
| --- | --- | --- | --- | --- | --- |
| *SR_closer: CON—BAS* | -0.0122 | [-0.0218, -0.0023] | -42.01 | [-65.71, -14.83] | 0.020 |
| *SR_closer: MAN—BAS* | 0.0032 | [-0.009, 0.0151] | 10.95 | [-27.79, 54.98] | 0.670 |
| *SR_closer: MAN—CON* | 0.0153 | [0.0024, 0.0274] | 90.79 | [5.9, 195.13] | 0.984 |
| *SR_further: CON—BAS* | 0.0059 | [-0.0095, 0.0239] | 22.083 | [-35.72, 89.18] | 0.731 |
| *SR_further: MAN—BAS* | 0.002 | [-0.0121, 0.0165] | 7.35 | [-41.72, 62.02] | 0.589 |
| *SR_further: MAN—CON* | -0.0041 | [-0.0234, 0.0159] | -12.51 | [-57.78, 48.65] | 0.365 |

| **Effect of size ratio:** | | |  |  |  |
| --- | --- | --- | --- | --- | --- |
| size.ratio_z | 0.0375 | [-0.2149, 0.2733] | - | - | 0.608 |

Bayesian pairwise contrasts derived from beta mixed-effects model: Proactive submissive avoidance (proportion of time visible) ~ treatment*size.ratio_z + (1|groupID). Contrasts represent posterior differences between treatments (marginalised over size.ratio_z) and between treatments within each size.ratio_z level.


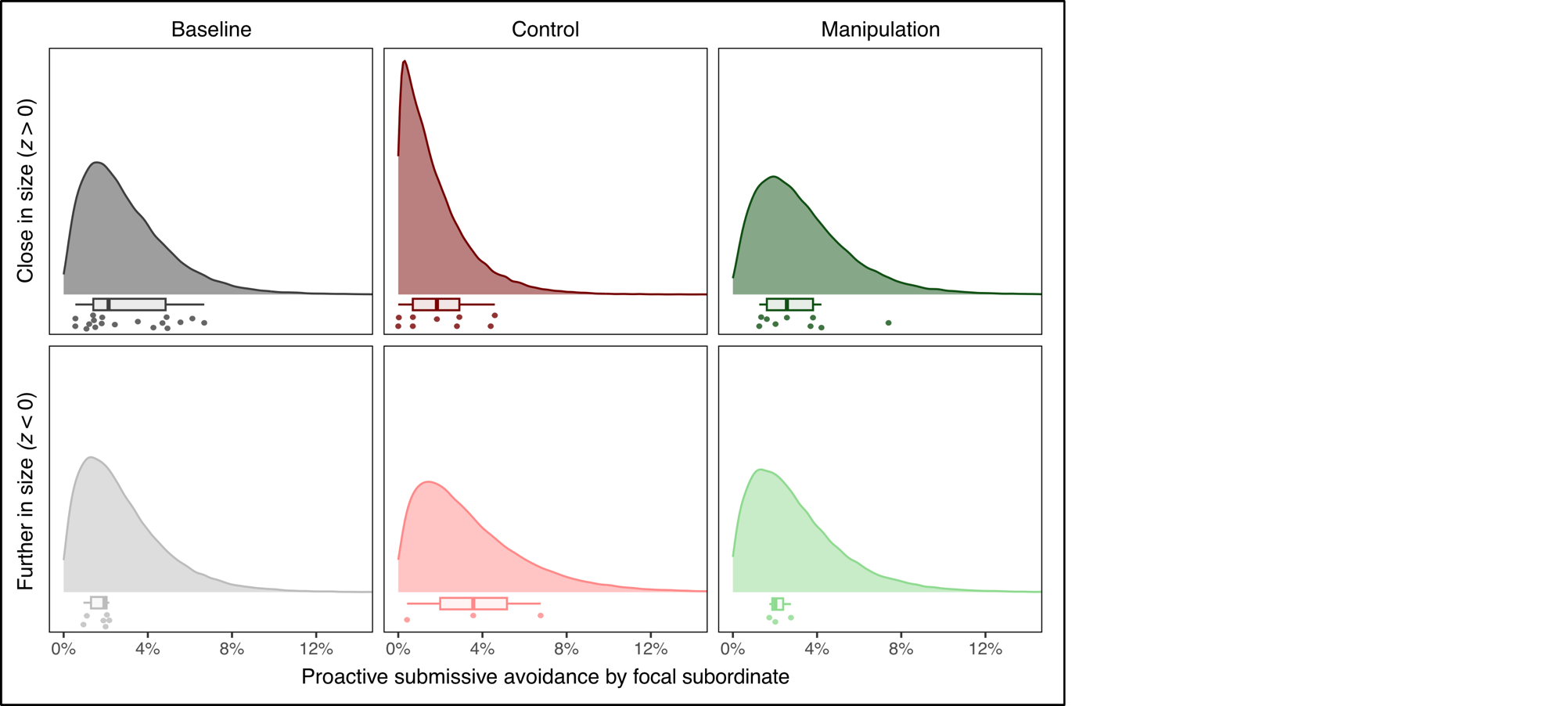


**Figure S12. Proactive submissive avoidance towards the focal subordinate across conditions (baseline, control, treatment) at each size ratio level (close in size, further in size) in *Amphiprion perideraion*.** Curves show posterior predicted distributions of avoidance (percentage of time visible) from the fitted model. Boxplots summarize observed values and points represent individual observations.

**Table S13**:

Treatment effects on proactive submissive avoidance by focal subordinate in *Amphiprion clarkii*.

| **Contrast/Term** | **Median Δ** | **89% HDI (Δ)** | **Median %** | **89% HDI (%)** | **Pr(Δ>0)** |
| --- | --- | --- | --- | --- | --- |
| **Treatment comparisons:** |  |  |  |  |  |
| *CON—BAS* | 0.0029 | [-0.0034, 0.0096] | 29.1703 | [-33.38, 102.32] | 0.764 |
| *MAN—BAS* | -0.0012 | [-0.0066, 0.0042] | -12.5016 | [-58.26, 40.90] | 0.354 |
| *MAN—CON* | -0.0041 | [-0.0114, 0.0031] | -31.9104 | [-69.22, 20.13] | 0.174 |

Bayesian pairwise contrasts derived from a beta mixed-effects model: Proactive submissive displays (proportion of time visible) ~ treatment + (1|groupID). Contrasts represent posterior differences between treatments.


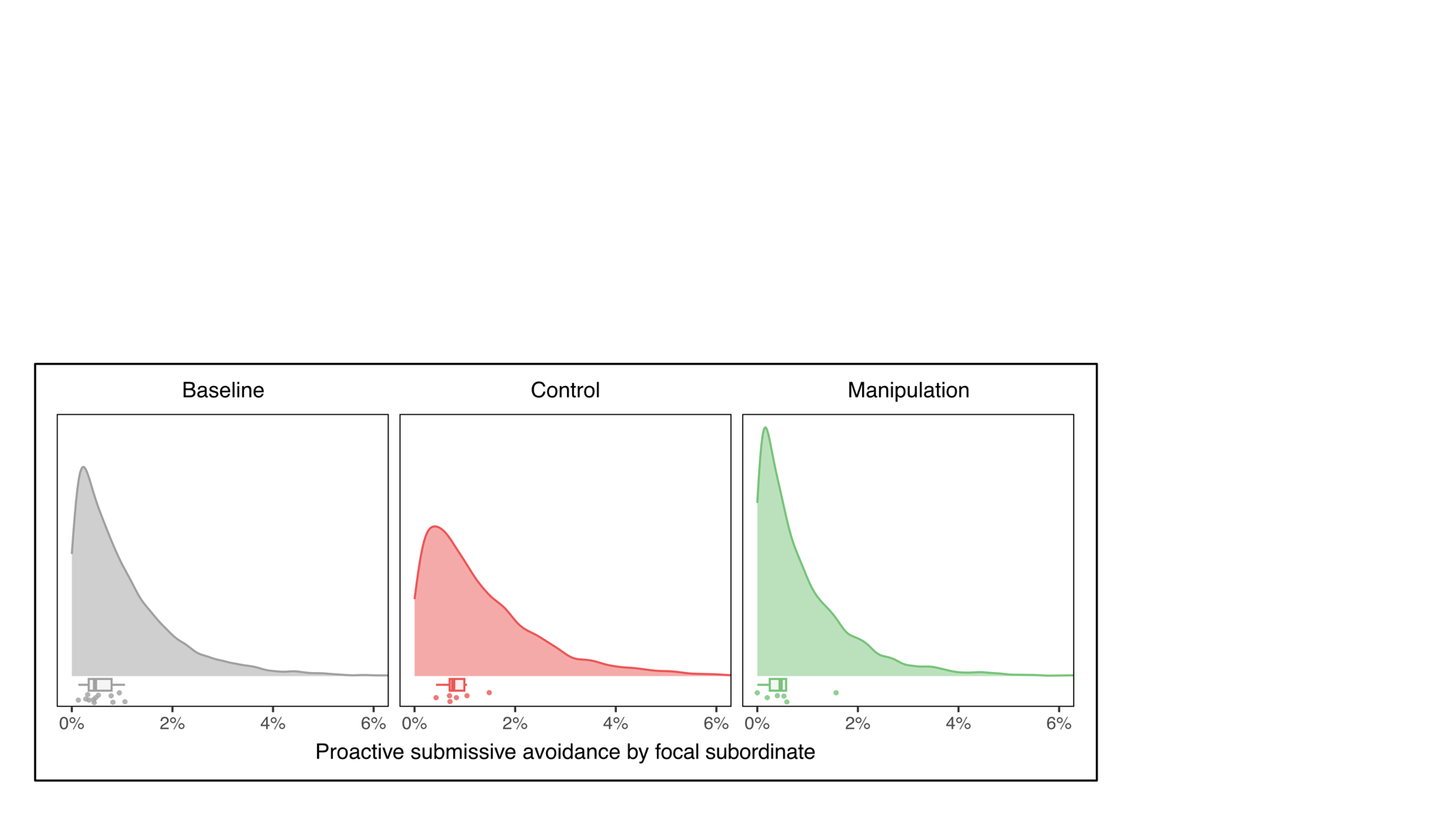


**Figure S13. Proactive submissive avoidance towards the focal subordinate across conditions (baseline, control, treatment) in *Amphiprion clarkii*.** Curves show posterior predicted distributions of avoidance (percentage of time visible) from the fitted model. Boxplots summarize observed values and points represent individual observations.

**Table S14**:

Treatment effects on territory defence by focal subordinate in *Amphiprion percula*.

| **Contrast/Term** | **Median Δ** | **89% HDI (Δ)** | **Median %** | **89% HDI (%)** | **Pr(Δ>0)** |
| --- | --- | --- | --- | --- | --- |
| **Treatment comparisons:** |  |  |  |  |  |
| *CON—BAS* | 0.0009 | [-0.0061, 0.0081] | 14.46 | [-68.6, 118.03] | 0.593 |
| *MAN—BAS* | -0.0013 | [-0.0084, 0.0057] | -19.92 | [-83.97, 72.20] | 0.353 |
| *MAN—CON* | -0.0021 | [-0.011, 0.0057] | -29.48 | [-89.68, 66.44] | 0.304 |

| **Treatment comparisons within each size ratio level:** | | |  |  |  |
| --- | --- | --- | --- | --- | --- |
| *SR_closer: CON—BAS* | 0.002 | [-0.0089, 0.0127] | 33.68 | [-80.98, 207.75] | 0.663 |
| *SR_closer: MAN—BAS* | -0.0014 | [-0.0105, 0.0067] | -25.15 | [-92.44, 78.71] | 0.347 |
| *SR_closer: MAN—CON* | -0.0035 | [-0.0154, 0.0057] | -43.55 | [-94.48, 52.24] | 0.235 |
| *SR_further: CON—BAS* | -0.0006 | [-0.0064, 0.0058] | -9.70 | [-75.8, 83.26] | 0.427 |
| *SR_further: MAN—BAS* | -0.0015 | [-0.0091, 0.0056] | -26.96 | [-90.6, 78.74] | 0.332 |
| *SR_further: MAN—CON* | -0.001 | [-0.0095, 0.0078] | -19.28 | [-93.6, 130.81] | 0.397 |

| **Effect of size ratio:** | | |  |  |  |
| --- | --- | --- | --- | --- | --- |
| size.ratio_*z* | 0.3546 | [-0.1555, 0.8605] | - | - | 0.878 |

Bayesian pairwise contrasts derived from zero inflated beta mixed-effects model: Territory defence (proportion of time visible) ~ treatment*size.ratio_z + (1|groupID). Contrasts represent posterior differences between treatments (marginalised over size.ratio_z) and between treatments within each size.ratio_z level.


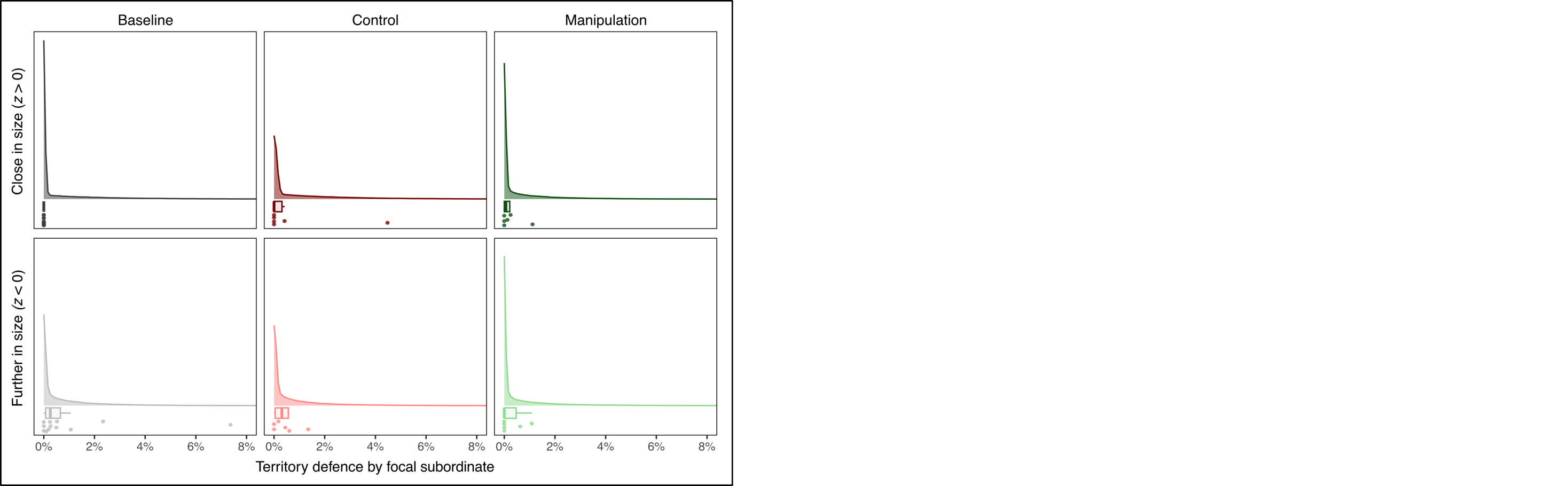


**Figure S14. Territory defence by the focal subordinate across conditions (baseline, control, treatment) at each size ratio level (close in size, further in size) in *Amphiprion percula*.** Curves show posterior predicted distributions of defence (percentage of time visible) from the fitted model. Boxplots summarize observed values and points represent individual observations.

**Table S15**:

Treatment effects on territory defence by focal subordinate in *Amphiprion perideraion*.

| **Contrast/Term** | **Median Δ** | **89% HDI (Δ)** | **Median %** | **89% HDI (%)** | **Pr(Δ>0)** |
| --- | --- | --- | --- | --- | --- |
| **Treatment comparisons:** |  |  |  |  |  |
| *CON—BAS* | -0.0001 | [-0.0033, 0.0036] | -2.79 | [-73.67, 91.09] | 0.478 |
| *MAN—BAS* | -0.0002 | [-0.0034, 0.0038] | -4.21 | [-78.15, 91.57] | 0.466 |
| *MAN—CON* | -0.0001 | [-0.0047, 0.0046] | -2.33 | [-85.04, 141.35] | 0.487 |

| **Treatment comparisons within each size ratio level:** | | |  |  |  |
| --- | --- | --- | --- | --- | --- |
| *SR_closer: CON—BAS* | 0.0003 | [-0.003, 0.0041] | 10.40 | [-74.05, 137.76] | 0.566 |
| *SR_closer: MAN—BAS* | -0.0009 | [-0.0041, 0.0018] | -33.24 | [-90.33, 51.32] | 0.276 |
| *SR_closer: MAN—CON* | -0.0012 | [-0.0055, 0.0024] | -39.26 | [-94.95, 62.05] | 0.262 |
| *SR_further: CON—BAS* | -0.0006 | [-0.0044, 0.004] | -15.50 | [-82.27, 90.09] | 0.396 |
| *SR_further: MAN—BAS* | 0.0000 | [-0.0039, 0.005] | 1.20 | [-83.13, 114.22] | 0.508 |
| *SR_further: MAN—CON* | 0.0006 | [-0.0052, 0.0065] | 18.60 | [-95.29, 235.39] | 0.580 |

| **Effect of size ratio:** | | |  |  |  |
| --- | --- | --- | --- | --- | --- |
| size.ratio_*z* | 0.1342 | [-0.2056, 0.4878] | - | - | 0.737 |

Bayesian pairwise contrasts derived from zero inflated beta mixed-effects model: Territory defence (proportion of time visible) ~ treatment*size.ratio_z + (1|groupID). Contrasts represent posterior differences between treatments (marginalised over size.ratio_z) and between treatments within each size.ratio_z level.


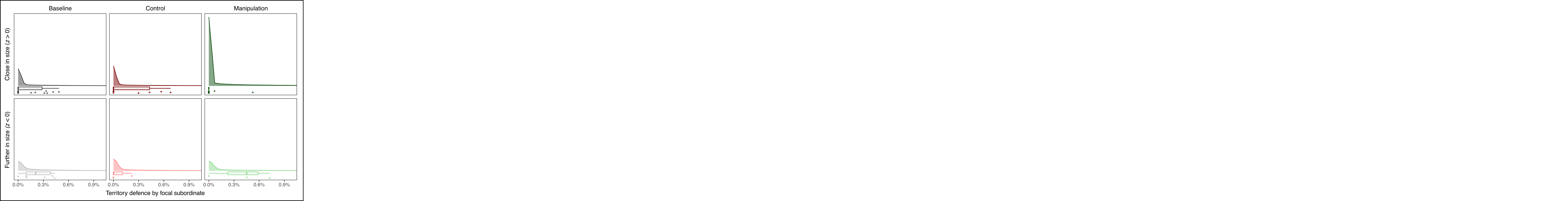


**Figure S15. Territory defence by the focal subordinate across conditions (baseline, control, treatment) at each size ratio level (close in size, further in size) in *Amphiprion perideraion*.** Curves show posterior predicted distributions of defence (percentage of time visible) from the fitted model. Boxplots summarize observed values and points represent individual observations.

**Table S16**:

Treatment effects on territory defence by focal subordinate in *Amphiprion clarkii*.

| **Contrast/Term** | **Median Δ** | **89% HDI (Δ)** | **Median %** | **89% HDI (%)** | **Pr(Δ>0)** |
| --- | --- | --- | --- | --- | --- |
| **Treatment comparisons:** |  |  |  |  |  |
| *CON—BAS* | 0.001 | [-0.0077, 0.0135] | 12.22 | [-81.32, 131.69] | 0.574 |
| *MAN—BAS* | 0.0072 | [-0.0043, 0.0218] | 82.28 | [-53.69, 243.30] | 0.861 |
| *MAN—CON* | 0.0056 | [-0.0103, 0.023] | 62.39 | [-76.41, 324.55] | 0.752 |

Bayesian pairwise contrasts derived from zero-inflated beta mixed-effects model: Territory defence (proportion of time visible) ~ treatment + (1|groupID). Contrasts represent posterior differences between treatments.


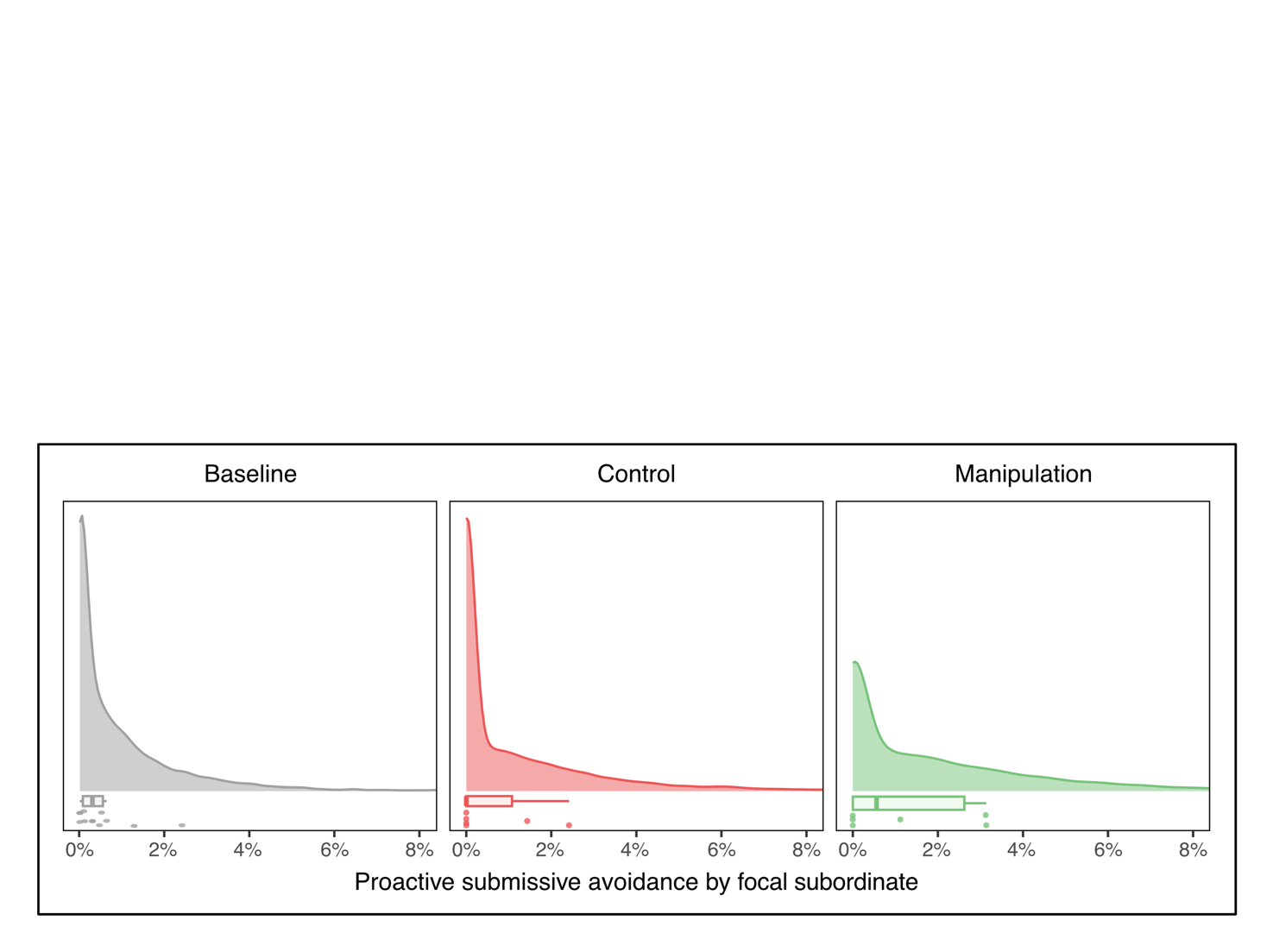


**Figure S16. Territory defence by the focal subordinate across conditions (baseline, control, treatment) in *Amphiprion clarkii*.** Curves show posterior predicted distributions of defence (percentage of time visible) from the fitted model. Boxplots summarize observed values and points represent individual observations.

**Table S17**:

Treatment effects on anemone maintenance by focal subordinate in *Amphiprion percula*.

| **Contrast/Term** | **Median Δ** | **89% HDI (Δ)** | **Median %** | **89% HDI (%)** | **Pr(Δ>0)** |
| --- | --- | --- | --- | --- | --- |
| **Treatment comparisons:** |  |  |  |  |  |
| *CON—BAS* | -0.0026 | [-0.0181, 0.012] | -4.74 | [-30.75, 22.8503] | 0.393 |
| *MAN—BAS* | -0.0063 | [-0.0211, 0.0081] | -11.72 | [-36, 14.731] | 0.241 |
| *MAN—CON* | -0.0036 | [-0.0214, 0.0128] | -7.29 | [-36.9, 26.69] | 0.361 |

| **Treatment comparisons within each size ratio level:** | | |  |  |  |
| --- | --- | --- | --- | --- | --- |
| *SR_closer: CON—BAS* | -0.006 | [-0.0273, 0.0171] | -9.127 | [-38.45, 22.8368] | 0.326 |
| *SR_closer: MAN—BAS* | -0.0138 | [-0.0343, 0.0074] | -20.989 | [-47.64, 6.9214] | 0.131 |
| *SR_closer: MAN—CON* | -0.008 | [-0.033, 0.0152] | -13.223 | [-48.64, 22.9956] | 0.283 |
| *SR_further: CON—BAS* | 0.0004 | [-0.0168, 0.0164] | 1.085 | [-39.82, 42.2683] | 0.519 |
| *SR_further: MAN—BAS* | 0.0015 | [-0.016, 0.0179] | 3.854 | [-35.61, 47.4208] | 0.559 |
| *SR_further: MAN—CON* | 0.0011 | [-0.0197, 0.0194] | 2.940 | [-43.66, 53.5317] | 0.540 |

| **Effect of size ratio:** | | |  |  |  |
| --- | --- | --- | --- | --- | --- |
| size.ratio_*z* | 0.3194 | [0.0306, 0.6105] | - | - | 0.958 |

Bayesian pairwise contrasts derived from zero inflated beta mixed-effects model: Anemone maintenance (proportion of time visible) ~ treatment*size.ratio_z + (1|groupID). Contrasts represent posterior differences between treatments (marginalised over size.ratio_z) and between treatments within each size.ratio_z level.


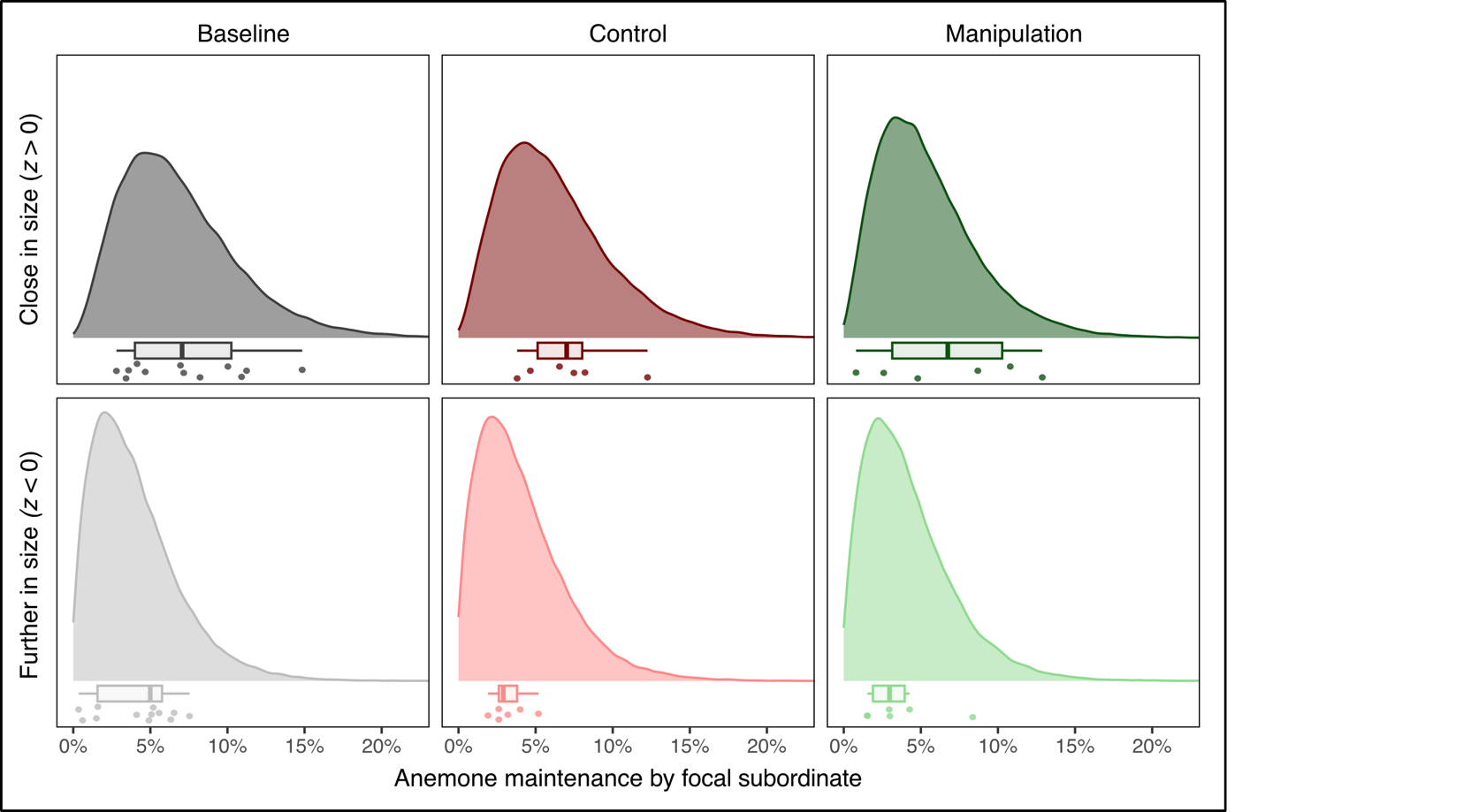


**Figure S17. Anemone maintenance by the focal subordinate across conditions (baseline, control, treatment) at each size ratio level (close in size, further in size) in *Amphiprion percula*.** Curves show posterior predicted distributions of maintenance (percentage of time visible) from the fitted model. Boxplots summarize observed values and points represent individual observations.

**Table S18**:

Treatment effects on anemone maintenance by focal subordinate in *Amphiprion perideraion*.

| **Contrast/Term** | **Median Δ** | **89% HDI (Δ)** | **Median %** | **89% HDI (%)** | **Pr(Δ>0)** |
| --- | --- | --- | --- | --- | --- |
| **Treatment comparisons:** |  |  |  |  |  |
| *CON—BAS* | -0.0021 | [-0.007, 0.0029] | -19.83 | [-57.43, 23.4816] | 0.238 |
| *MAN—BAS* | -0.0032 | [-0.0076, 0.0016] | -30.18 | [-63.71, 8.3354] | 0.130 |
| *MAN—CON* | -0.0011 | [-0.0064, 0.004] | -12.52 | [-64.31, 45.4607] | 0.370 |

| **Treatment comparisons within each size ratio level:** | | |  |  |  |
| --- | --- | --- | --- | --- | --- |
| *SR_closer: CON—BAS* | -0.0018 | [-0.0093, 0.0055] | -12.652 | [-58.09, 36.851] | 0.339 |
| *SR_closer: MAN—BAS* | -0.0029 | [-0.0102, 0.0045] | -20.032 | [-61.57, 29.11] | 0.265 |
| *SR_closer: MAN—CON* | -0.001 | [-0.0098, 0.0075] | -7.8143 | [-62.22, 63.476] | 0.426 |
| *SR_further: CON—BAS* | -0.0027 | [-0.008, 0.0026] | -33.8651 | [-78.27, 23.3421] | 0.195 |
| *SR_further: MAN—BAS* | -0.0039 | [-0.0088, 5e-04] | -50.0283 | [-84.3, -7.2404] | 0.078 |
| *SR_further: MAN—CON* | -0.0012 | [-0.0068, 0.0038] | -23.9291 | [-82.55, 68.5079] | 0.331 |

| **Effect of size ratio:** | | |  |  |  |
| --- | --- | --- | --- | --- | --- |
| size.ratio_*z* | 0.2850 | [-0.336, 0.5962] | - | - | 0.933 |

Bayesian pairwise contrasts derived from zero inflated beta mixed-effects model: Anemone maintenance (proportion of time visible) ~ treatment*size.ratio_z + (1|groupID). Contrasts represent posterior differences between treatments (marginalised over size.ratio_z) and between treatments within each size.ratio_z level.

**
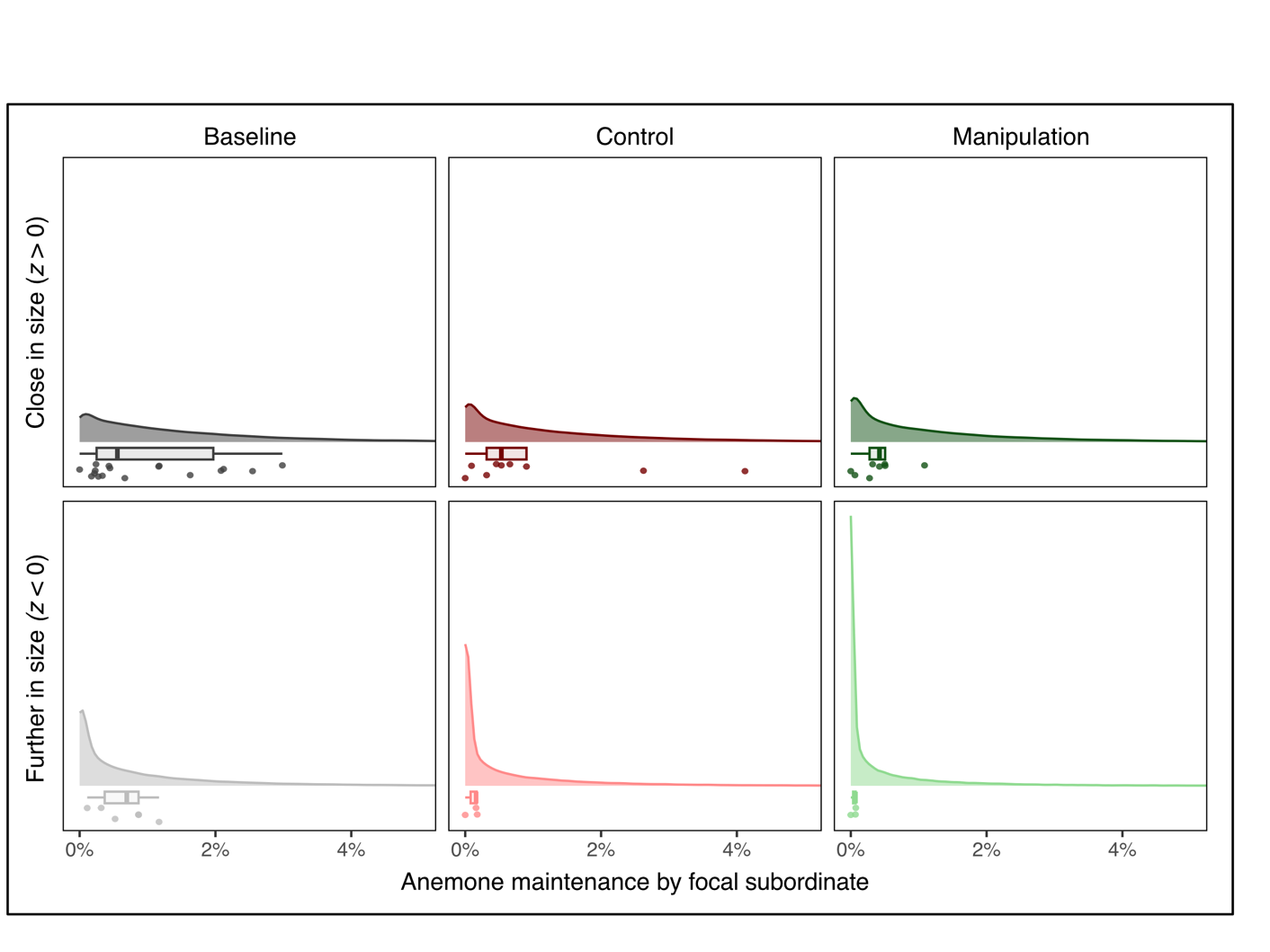
**

**Figure S18. Anemone maintenance by the focal subordinate across conditions (baseline, control, treatment) at each size ratio level (close in size, further in size) in *Amphiprion perideraion*.** Curves show posterior predicted distributions of maintenance (percentage of time visible) from the fitted model. Boxplots summarize observed values and points represent individual observations.

**Table S19**:

Treatment effects on anemone maintenance by focal subordinate in *Amphiprion clarkii*.

| **Contrast/Term** | **Median Δ** | **89% HDI (Δ)** | **Median %** | **89% HDI (%)** | **Pr(Δ>0)** |
| --- | --- | --- | --- | --- | --- |
| **Treatment comparisons:** |  |  |  |  |  |
| *CON—BAS* | 0.002 | [-0.0043, 0.0094] | 35.13 | [-65.8, 166.32] | 0.704 |
| *MAN—BAS* | 0.0093 | [0.0006, 0.0191] | 165.67 | [-5.05, 392.16] | 0.976 |
| *MAN—CON* | 0.0073 | [-0.0033, 0.0187] | 96.33 | [-47.35, 326.67] | 0.877 |

Bayesian pairwise contrasts derived from zero-inflated beta mixed-effects model: Anemone maintenance (proportion of time visible) ~ treatment + (1|groupID). Contrasts represent posterior differences between treatments.

**
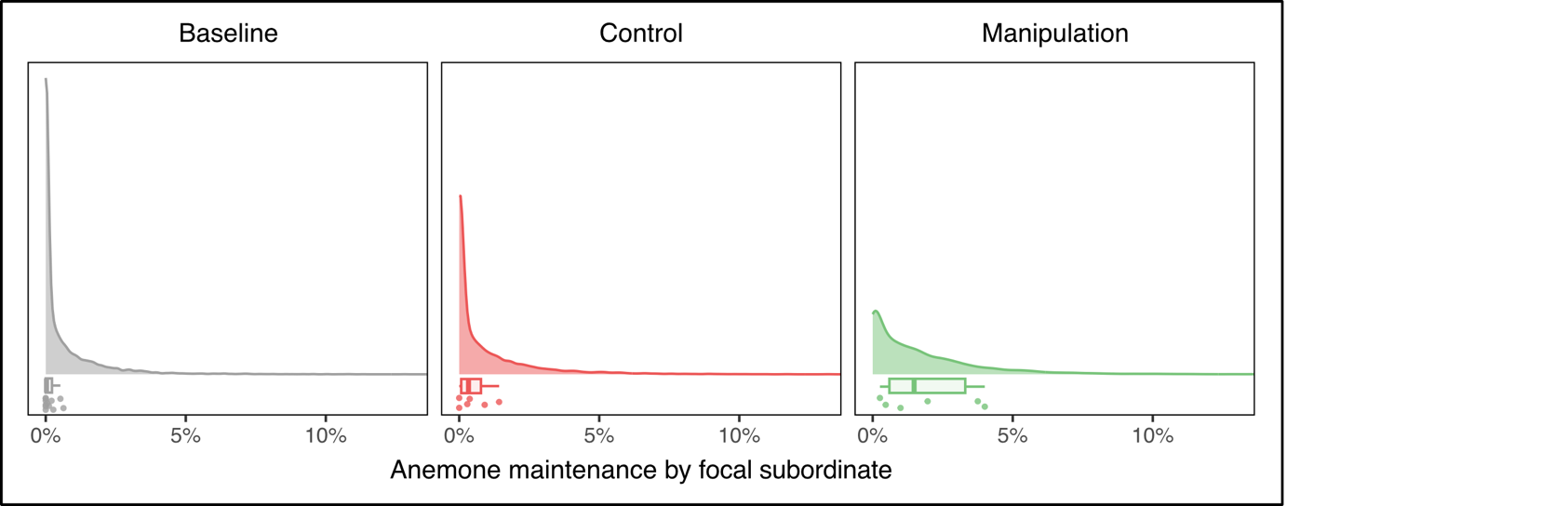
**

**Figure S19. Anemone maintenance by the focal subordinate across conditions (baseline, control, treatment) in *Amphiprion clarkii*.** Curves show posterior predicted distributions of maintenance (percentage of time visible) from the fitted model. Boxplots summarize observed values and points represent individual observations.
